# A Two-Stage Cascaded Regression Framework for Automatic Facial Acupoint Localization in Infrared Thermal Images

**DOI:** 10.3390/s26144498

**Published:** 2026-07-15

**Authors:** Jiahao Li, Xingcheng Ming, Ying Zeng, Ruifeng Yang, Jin Tian, Fu Niu

**Affiliations:** Systems Engineering Institute, Academy of Military Sciences, PLA, Beijing 100091, China

**Keywords:** acupoint localization, infrared thermal image, Traditional Chinese Medicine, geometric constraints, keypoints detection, object detection

## Abstract

**Highlights:**

**What are the main findings?**

- We constructed a multi-pose thermal facial dataset and mathematically translated classical TCM anatomical rules into five explicit geometric constraint models.
- We developed T2FAL, a two-stage framework that separates thermal face detection and pose routing from pose-specific acupoint localization.

**What are the implications of the main findings?**

- The digitization of classical TCM measurement rules offers a standardized, objective framework that reduces the subjectivity and inter-practitioner deviations inherent in traditional manual localization.
- This two-stage multi-pose framework performs view-specific localization for frontal and profile faces and is intended to reduce coordinate variation associated with pose and perspective. By providing standardized facial acupoint coordinates, the framework may support downstream thermal-region analysis and provide a technical basis for future research on automated body constitution identification, early disease warning, clinical diagnosis, therapeutic evaluation, and primary healthcare management.

**Abstract:**

Infrared thermal imaging offers objective physiological insights for Traditional Chinese Medicine (TCM), yet automated acupoint localization struggles with low texture and extreme pose variations. To address this, we constructed a multi-pose thermal facial dataset using digitized bone-proportional measurements and TCM anatomical rules. We propose T2FAL, a two-stage cascaded regression framework explicitly decoupling macroscopic face detection from fine-grained acupoint localization. Stage 1 utilizes an improved YOLOv12m-based Thermal-Aware Face Detector—integrating ICAN_C2f, MixNeck, and TAF-IoU—to mitigate domain shifts and thermal noise. Stage 2 deploys pose-specific regressors incorporating Gated Feature-Conditioned Cascade Refinement (FCCR) and a Selective GeoDeriv module, mathematically translating anatomical rules into geometric constraints. Under the stated experimental protocol, Stage 1 processed images at 87.3 frames per second on an NVIDIA RTX 4090. For Stage 2, the final frontal- and profile-view configurations achieved mAP@50–95 values of 73.19% and 86.92%, with mean pixel errors of 1.986 and 3.109 pixels, respectively. These results support the feasibility of automated reference-coordinate localization on the datasets used. However, given the partial reliance of the annotations on image registration and geometric rules, the use of the cross-domain set for model selection, and the lack of clinical or diagnostic evaluation, further validation using independently generated expert annotations, a strictly held-out external test set, and clinically labeled data is required before clinical or diagnostic use.

## 1. Introduction

Globally, non-communicable diseases (NCDs) have become the leading causes of mortality and disability. With accelerating population aging, the associated disease burden continues to rise [1]. Many chronic conditions involve a prolonged subclinical phase before clinical symptoms manifest, during which metabolic or functional abnormalities have already occurred [2]. This pre-disease state highlights the importance of early intervention and provides supporting evidence for the Traditional Chinese Medicine (TCM) concept of preventive treatment [3]. This concept advocates a hierarchical prevention strategy of preventing disease before its onset and mitigating deterioration after illness. It aligns with contemporary principles of full-cycle chronic disease management [4]. The Theory of Meridians and Acupoints serves as the technical foundation for implementing this preventive framework. Acupoints are defined as specific sites where the vital energy and blood of the internal organs and meridians converge on the body surface. They function as targets for external therapies and as external manifestations of the body’s internal physiological state [5].

However, traditional localization methods long established in clinical practice, such as anatomical landmarks, bone-proportional measurements, finger-proportional measurements, and simple orientation techniques, rely on subjective judgment. These methods often produce large inter-practitioner deviations that can reach several centimeters or exhibit substantial proportional variations [6]. Such variability compromises the reproducibility of clinical efficacy and hinders evidence-based evaluation of acupuncture therapies [7]. There is therefore a clear need to develop objective and standardized automatic localization schemes through modern technical means such as deep learning. This development matters for overcoming the limitations of traditional methodologies and for advancing the modernization of TCM.

Computer vision-based automated acupoint localization has become an active research direction in the modernization of TCM. Current mainstream methods focus primarily on visible light (RGB) images. Their core approach uses deep learning to extract surface anatomical landmarks or generic human keypoints as geometric constraints. They then infer final acupoint coordinates via proportional interpolation or distance offsets guided by TCM theory [8,9,10,11,12,13]. Some researchers have adopted direct localization approaches that regress acupoint coordinates end-to-end, implemented through direct coordinate regression [14,15,16] or Gaussian heatmaps [17,18]. The Transformer-based AIR-Net model learns spatial correspondences between image pairs. It improves acupoint detection on the back of the hand across various skin tones and postures [19]. In lightweight design, the YOLOv8-ACU framework integrates the Efficient Channel Attention (ECA) mechanism and shows advantages in both accuracy and computational efficiency [20]. Binocular vision-based human acupoint localization systems have extended 2D localization into 3D space for precise recognition in clinical treatment scenarios [21]. Despite this progress, RGB-modality methods remain dependent on ambient lighting conditions. They capture only skin surface texture and fail to acquire information reflecting the functional states of subcutaneous tissues or the activity of vital energy and blood. Consequently, they struggle to elucidate the deeper physiological mechanisms of acupoints and their broader medical applications remain limited.

In contrast, infrared thermal imaging is a rapid, passive, and non-contact temperature detection technology. It acquires the temperature distribution across the human body surface in real time and remains fundamentally unaffected by ambient lighting. These properties confer advantages in real-time performance, safety, and robustness, leading to its widespread use across various medical domains [22]. Thermal imaging can reflect abnormal variations in human thermal radiation distribution. It can thereby capture acupoint sensitization triggered by internal organ dysfunction and enable an objective, visual representation of cold and heat information [23]. With continuous advances in uncooled infrared sensor technologies, low-cost portable thermal cameras now deliver high thermal sensitivity and good imaging quality. The resulting reduction in equipment costs has established a hardware foundation for broad medical application [24,25]. Automated acupoint localization technology based on thermal imaging can therefore combine the early-warning capabilities of functional imaging with the TCM concept of preventive treatment. It can support body constitution identification, disease prevention, diagnosis, evaluation, and the development of primary healthcare management systems.

However, the application of infrared thermal imaging to acupoint localization remains at an early stage. Existing research has relied predominantly on traditional image processing and rule-driven methods, while deep learning-based investigations are still limited. Early and some recent studies have employed traditional techniques such as corner and edge detection for acupoint region extraction and feature analysis [26,27,28,29]. Lian et al. proposed a method that uses infrared thermal imaging to assist in partially occluded face recognition. By coupling TCM finger-proportional measurement rules with a Backpropagation (BP) neural network, they established a mapping relationship between facial feature points and acupoint locations for auxiliary localization of facial acupoints [30]. Nevertheless, this approach combines artificial prior geometric constraints with shallow machine learning and depends strongly on facial proportional relationships and handcrafted feature design. Deep learning applications in this field remain confined to a few specific scenarios. For example, Xia et al. [31] combined the Single Shot MultiBox Detector (SSD) algorithm with Dlib keypoint detection to localize the Yintang, Cuanzhu, and Yuyao acupoints in thermal images to assist temperature measurement. However, this method localizes only a limited number of acupoints concentrated in the local forehead region. The field currently faces three main challenges. First, infrared thermal acupoint datasets are scarce, which severely restricts algorithm training and validation. Second, infrared thermal images lack texture and thermal diffusion effects produce blurred boundaries. Generic vision models therefore achieve relatively low localization accuracy on this low-feature modality [32]. Third, substantial posture variations and individual anatomical differences make traditional single-stage regression models prone to local optima. Without effective anatomical geometric constraints, some acupoints experience large coordinate shifts that lead to localization failure.

Inspired by top-down pose estimation architectures such as HRNet [33], RTMPose [34], and ViTPose [35], we propose the Two-Stage Thermal Facial Acupoint Localization (T2FAL) framework. The framework integrates classical TCM anatomical theory with image enhancement and advanced deep cascaded regression algorithms to address the inherent ambiguity of infrared thermal imagery.

To establish a data foundation, we constructed a multi-pose thermal facial acupoint dataset. We grounded the annotations in digital bone-proportional measurements. This fills a gap in the field and reduces annotation bias. Building on this dataset, the T2FAL framework implements an adaptive coarse-to-fine two-stage localization pipeline. In the first stage, we apply Contrast Limited Adaptive Histogram Equalization (CLAHE) for hotspot enhancement. We then deploy a Thermal-Aware Face Detector to overcome semantic noise, rapidly isolate the macroscopic facial Region of Interest (ROI), and extract pose priors. In the second stage, we couple a lightweight progressive regression network with a Gated Feature-Conditioned Cascade Refinement (Gated FCCR) module for dynamic sub-pixel optimization. Concurrently, we introduce a Selective Geometric Derivation (Selective GeoDeriv) mechanism. This mechanism translates human facial anatomy into explicit topological constraints to correct coordinate offsets induced by severe perspective distortions or boundary ambiguity.

Experimental validation shows that T2FAL establishes a mapping from fuzzy thermal fields to precise anatomical coordinates and improves localization stability under complex poses. The framework therefore addresses the localization layer required for subsequent thermal-zone feature extraction and clinical association studies; clinical screening and diagnosis remain tasks for future validation with appropriate clinical labels.

## 2. Materials and Methods

Given the absence of publicly available benchmark datasets in this field, we first constructed a multi-pose thermal imaging acupoint dataset. Based on this foundation, we describe the two cascaded stages in sequence according to the processing workflow: (1) the frontal and profile face detection stage, which automatically identifies facial orientation to reduce feature confusion; and (2) the acupoint detection stage, which performs precise acupoint coordinate regression under specific viewpoints.

### 2.1. Thermal Facial Acupoint Dataset Construction

To address the lack of thermal acupoint datasets, this study utilizes the Tufts Face Database (TFD) [36] and the Charlotte-ThermalFace (CTF) dataset [37]. We established a multi-pose thermal dataset of frontal and profile faces based on posture categorization criteria, standardized medical acupoint definitions, digital bone-proportional measurement baselines, and cross-modal registration and annotation strategies. The subsequent sections detail data source selection, preprocessing procedures, subset construction, data augmentation, and partitioning.

#### 2.1.1. Data Source and Preprocessing

To improve model generalization, we selected the open-source TFD and CTF datasets. We used the LabelMe tool [38] to annotate faces, acupoints, and indirect localization reference points uniformly. The two datasets complement each other. CTF emphasizes complex environmental conditions and contains over 10,000 images from 10 subjects across temperatures of 20.5 °C to 26.5 °C. Its main strengths are comprehensive metadata—including shooting distances (1.0 m to 6.6 m), 25 head postures, wind speed, humidity, and thermal sensation labels—together with 16-bit raw radiometric data that preserves pixel-level temperature values. TFD emphasizes demographic diversity and multi-modal correlations. It includes 113 subjects of varied demographics, captures multi-view data with a 9-camera semicircular array, and provides six paired modalities (thermal, visible light, and near-infrared).

During integration, we cleaned and filtered the original sources to improve annotation reliability while preserving realistic sample variation. From CTF, we selected 1030 high-resolution samples (e.g., 465 × 350, 450 × 400, and 400 × 350). From TFD, which follows a uniform acquisition protocol, we extracted three subsets: upper-body thermal images (TD-IR, 336 × 256), thermal faces (Thermal-faces-128 × 128), and visible-light faces (RGB-faces-128 × 128). Images with mild-to-moderate hair occlusion, eyeglasses, headscarves, motion blur, or locally weak thermal boundaries were retained when the facial contour, major facial organs, and target annotation regions remained identifiable. Samples were excluded when severe defocus, large missing facial regions, unrecognizable anatomical references, heavy occlusion of target regions, or extreme pose made reliable annotation impossible; this criterion led to the exclusion of three subjects with severe occlusions. In total, we selected 1504 images.

#### 2.1.2. Face Detection and Pose Classification Dataset

The public datasets provide only discrete angle labels or lack explicit posture annotations. Simple geometric angle division does not satisfy the requirements of infrared thermal imaging for optimal observation planes, which must minimize radiation distortion and maximize feature exposure. We therefore developed a three-tier classification criterion based on the practical needs of medical acupoint localization. The criterion integrates acupoint visibility, physical thermometric characteristics, and geometric area ratios to define frontal and profile faces.

Criterion 1 is based on visibility constraints of anatomical keypoints. We used the LI20 (Yingxiang) acupoint, which shows large depth variations at the nasolabial fold, as an anchor. A face is classified as frontal when both bilateral LI20 points are visible without occlusion by the nasal projection. This ensures feature integrity in the central region (e.g., EX-HN3 (Yintang), GV26 (Renzhong)), where facial symmetry mapping applies. If only one side is visible or the distal end is occluded by the nasal contour while lateral acupoints such as TE21 (Ermen), SI19 (Tinggong), and GB2 (Tinghui) remain clearly exposed, the image is classified as profile. This distinction matters because the 16 lateral facial acupoints do not follow the same symmetry mapping as frontal points and require global spatial reasoning.

Criterion 2 follows Lambert’s Cosine Law for infrared imaging to minimize emissivity attenuation and thermometric distortion caused by large observation angles. A frontal face is defined as one in which the normal vector of the facial T-zone is approximately parallel to the camera optical axis (<30°). This preserves thermal accuracy of the forehead and inner cheeks. For profile faces, the normal vectors of the lateral cheek and preauricular regions must face the camera directly to capture maximal radiant energy.

Criterion 3 introduces a quantitative geometric metric bounded by the outer canthus. By calculating the area ratio between the region from the outer canthus to the nasal tip ($S_{\mathrm{inner}}$) and the region from the outer canthus to the auricle ($S_{\mathrm{outer}}$), this metric assists in determining the shift in the facial center of gravity. A frontal face predominantly exhibits a larger $S_{\mathrm{inner}}$, whereas a profile face demonstrates a significant increase in $S_{\mathrm{outer}}\;$ alongside complete auricular features.

We applied these three criteria to produce standardized binary bounding-box annotations. The ROI extends vertically from hairline to chin and horizontally covers both ears (frontal) or the area from nasal tip to one ear (profile). For CTF, which contains complex postures with varying pitch, yaw, and roll, we used a hierarchical decision process: first screen by facial vector orientation; for ambiguous samples, check acupoint visibility and completeness; finally apply the outer-canthus area ratio for the final decision. For TFD, which has nine discrete angles in the A-series and five expressions in the E-series, we performed high-precision classification directly from acupoint visibility and vector orientation, because A-4 and E-series represent standard frontal faces while A-0 and A-8 represent standard profile faces with small pitch and yaw.

**Figure 1:**
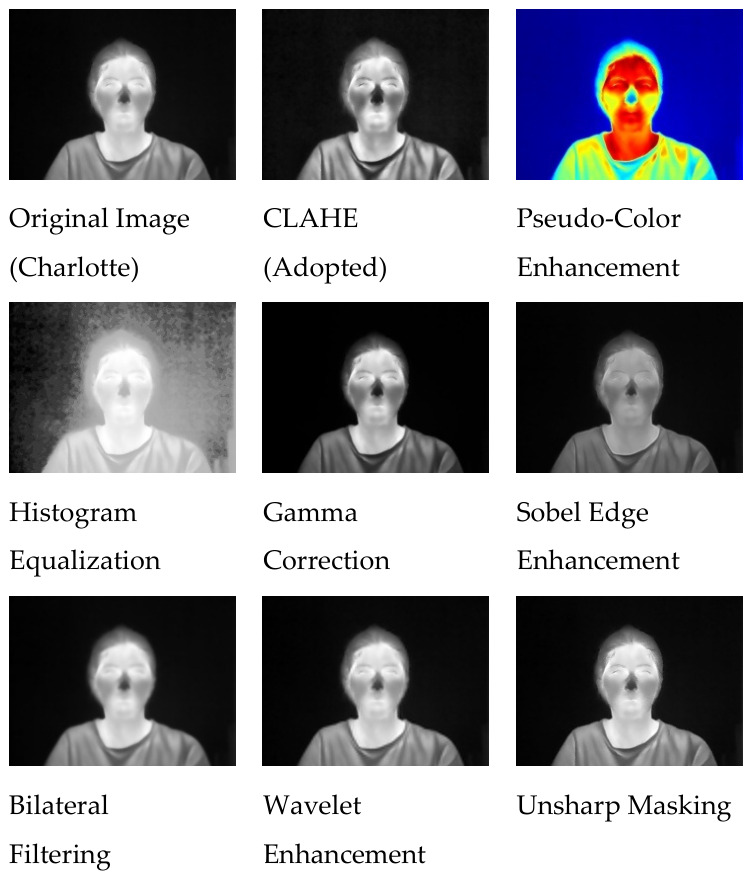
Comparison of different image enhancement methods on the Charlotte dataset.

To overcome low contrast and ambiguous boundaries in thermal infrared images and to improve cross-domain generalization of the first-stage face detection and frontal/profile classification models, we applied targeted data augmentation. After comparing several classical enhancement methods, we selected Contrast Limited Adaptive Histogram Equalization (CLAHE) [39]. As shown in Figure 1, CLAHE improves local contrast in facial regions more effectively than the other tested methods and better highlights local textures and temperature gradients.

To simulate large posture changes in real environments and reduce overfitting risk, we performed geometric augmentation with in-plane random rotations and horizontal mirror flipping. We updated the corresponding facial bounding boxes synchronously to preserve spatial integrity. These transformations expanded the training samples to four times the original number and balanced the distribution between frontal and profile views. They also encouraged the detection network to learn rotational and flip invariance. After augmentation, we completed construction of the dataset for the first-stage detection task (Table 1).

#### 2.1.3. Pose-Specific Acupoint Localization Dataset

We defined the nomenclatures and anatomical locations of all acupoints according to the GB/T 12346-2021 standard [40]. For facial extra-meridian points outside the fourteen regular meridians (e.g., EX-HN3 (Yintang) and EX-HN5 (Taiyang)), we applied supplementary definitions from the GB/T 40997-2021 [41] and World Health Organization (WHO) standards [42]. We then corroborated these definitions with the GB/T 22163-2008 2D reference atlas [43] to map medical textual descriptions to precise image pixel coordinates.

To address texture degradation and perspective distortion in 2D thermal projections, we formulated independent Frontal View and Profile View classification systems. The Frontal View system contains 17 acupoint categories along the facial midline and central regions (e.g., EX-HN3 (Yintang), BL2 (Cuanzhu), GB14 (Yangbai), LI20 (Yingxiang), and GV26 (Renzhong)). It relies on central anatomical landmarks (canthi, superciliary arches, nasal alae, mouth corners) to model global topological relationships and bilateral symmetry. The Profile View system contains 11 acupoint categories in the periauricular, temporal, and mandibular regions (e.g., TE23 (Sizhukong), SI19 (Tinggong), ST6 (Jiache), and EX-HN5 (Taiyang)). It models lateral landmarks (auricle, zygomatic arch, mandibular angle) to mitigate recognition ambiguity and coordinate overlap for periauricular acupoints.

We developed a digital transformation algorithm that maps the TCM bone-proportional measurement method onto image geometric features. To account for structural anisotropy and thermal imaging discrepancies, we established distinct horizontal and vertical digital baselines based on classical TCM theory and thermal physical characteristics.

For the horizontal bone-proportional measurement baseline, we followed the definitions in the WHO Standard Acupuncture Point Locations in the Western Pacific. According to the localization descriptions and illustrations of the GB15 (Toulinqi) acupoint, the horizontal distance from the anterior median line to the ST8 (Touwei) acupoint equals 4.5 cun. The vertical line directly above the pupil intersects the midpoint of the horizontal segment connecting the anterior median line and ST8 (Touwei). It follows that the horizontal physiological distance between the centers of the bilateral pupils equals 4.5 horizontal cun. We therefore define the horizontal unit baseline as:

(1) $$1\;{H-Cun}_{frontal}=\frac{|x_{Pupil\_R}-x_{Pupil\_L}|}{4.5},$$

where $x_{\mathrm{Pupil}\_R}\;$ and $x_{\mathrm{Pupil}\_L}\;\;$ represent the x-coordinates of the right and left pupil centers on the image horizontal axis, respectively.

Selecting the bilateral pupillary distance as the horizontal baseline is supported by authoritative international medical standards. Anatomically, the ocular region (excluding natural eyelid blinking) shows high spatial stability because it is rarely distorted by facial expression muscles. Under thermal infrared imaging, the eyes and surrounding skin tissues exhibit distinct temperature gradient boundaries, which provide reliable features for automated pupil center extraction. In contrast, the ST8 (Touwei) acupoint lacks salient thermal signatures and is frequently occluded by the hairline, making it unsuitable for automated keypoint extraction.

For the vertical bone-proportional measurement baseline, we adopted the “iddle Third Axis Method.” This method follows the classical TCM “Three Facial Thirds” (San Ting) theory, which divides the face vertically into three approximately equal segments from the anterior hairline to the menton, demarcated by EX-HN3 (Yintang) and the nasal tip (GV25 (Suliao)). We selected the middle third region along the vertical facial midline as the vertical unit baseline because it offers greater imaging stability in thermal maps. The calculation formula is

(2) $$1\;\;V-\mathrm{Cun}=\frac{\left|y_{NoseTip}-y_{EX-HN3}\right|}{3},$$

where $y_{NoseTip}$ represents the y-coordinate of the nasal tip, and $y_{EX-HN3}$ is the y-coordinate of the EX-HN3 acupoint.

The upper third is often occluded by frontal hair, and the lower third is susceptible to thermal disturbances from nasal and oral airflow as well as blurred mentocervical boundaries caused by submental fat. The middle third (from EX-HN3 to the subnasale) possesses stable osseous structure. In thermal maps, the nasal tip appears as a low-radiance cold zone that forms a clear temperature gradient with surrounding skin and supplies distinct edge features.

Because distance and angular differences exist between frontal and profile captures, applying the frontal baseline is inappropriate. The profile region primarily requires horizontal cun measurements, and target acupoints such as GB1 (Tongziliao) lie around the eyes. We therefore adopted a local plane approximation strategy that assumes the eyeball and periorbital acupoints reside within the same local 2D plane. When the face rotates within a limited yaw angle range, the relative scaling ratio between feature points in this plane remains consistent.

We selected the distance between the inner and outer canthi of a single eye (palpebral fissure width) as the horizontal measurement baseline for the profile view. This feature provides robust detection and stable spatial relationships. To assign the corresponding cun value, we performed a mathematical derivation based on the neoclassical “Rule of Fifths.” Physiologically, the horizontal distance from the anterior median line to the pupil center equals 2.25 horizontal cun. According to the Rule of Fifths approximation, the distances from the median line to the inner canthus and from the inner canthus to the pupil center each occupy approximately 0.5 palpebral fissure widths. Consequently, the distance from the median line to the pupil center equals one full palpebral fissure width. We therefore deduce that the physical width from the inner canthus to the outer canthus equals approximately 2.25 horizontal cun. The profile horizontal unit baseline is defined as:

(3) $${1\;H-Cun}_{lateral}=\frac{|x_{LC}-x_{IC}|}{2.25},$$

where $x_{LC}\;$ and $x_{IC}\;$ represent the x-coordinates of the outer (lateral) and inner (medial) canthi, respectively, on the visible side of the profile image.

This dynamic baseline maintains mathematical consistency between frontal and profile dimension definitions and compensates for perspective distortion caused by head rotation. Using the established digital bone-proportional measurement baselines, we formalized the localization logic for the 28 categories of facial acupoints into five geometric constraint models.

The first category is the Direct Coordinate Mapping Method. It targets acupoints with prominent anatomical features that are distinguishable in thermal images (e.g., BL2, EX-HN8 (Shangyingxiang), GB2) and acupoints whose traditional localization relies on tactile palpation of underlying structures (e.g., GB3 (Shangguan), ST7 (Xiaguan), ST6, ST5 (Daying)). The method directly adopts the coordinates regressed by the fine-grained point regression network as the final acupoint locations.

The second category is the Decoupled Linear Interpolation Method. Rooted in TCM bone-proportional measurement theory, it transforms acupoint localization into a linear constraint problem with independent horizontal and vertical coordinates. It applies to acupoints with regular proportions (e.g., EX-HN3) and to complex acupoints that require cross-feature alignment (e.g., EX-HN4). The coordinate calculation formula is

(4) $$\{\begin{array}{ccccc} & x_{target} & =x_{i}+\alpha_{x}(x_{j}-x_{i}) & & \\ & y_{target} & =y_{m}+\alpha_{y}(y_{n}-y_{m}), & & \end{array}$$

where $x_{i},x_{j}$ and $y_{m},y_{n}$ represent the coordinates of the horizontal and vertical anatomical reference points, respectively, and $\alpha_{x},\alpha_{y}\in [0,\;1]\;$ are the corresponding interpolation coefficients. Taking EX-HN4 as an example, its horizontal localization is based on the description “vertically aligned with the pupil”; thus, the pupil center is selected as the primary reference point ($x_{i}=x_{pupil}$), and $\alpha_{x}=0\;$ is set to achieve vertical alignment. For vertical localization, based on the eyebrow’s trajectory, the medial end of the eyebrow (BL2, $y_{m}=y_{BL2}$) and the eyebrow peak ($y_{n}=y_{Peak}$) are selected as reference points, and $\alpha_{y}=0.5\;$ is set, designating the midpoint of the vertical segment connecting them as the target coordinate $y_{target}$.

The third category is the Dual-Line Orthogonal Constraint Method. This method employs point-direction geometric modeling, locking onto the acupoint by intersecting two lines that each pass through an independent keypoint and possess a specific direction vector. To maintain mathematical consistency, the superscript k∈{F,P}  is defined to represent the frontal and profile views, respectively, while d∈{v(up,down),h(lat,med)} denotes the vertical and horizontal constraints. Under this framework, any constraint line $L_{d\;}^{k}$ is uniquely determined by an independent anatomical anchor point $P_{d}^{k}\;$ and a direction vector ${\vec{v}}_{d}^{k}$.

For the frontal view, this model is applicable to the localization of ST3 (Juliao) and LI19 (Kouheliao). The horizontal constraint $L_{h}^{F}\;$ is established by the anchor point $P_{h}^{F}$ and the direction vector ${\vec{v}}_{h\;}^{F}$ (the line connecting the bilateral outer canthi). The vertical constraint $L_{v}^{F}\;$ passes through the reference point $P_{v}^{F}$, and its direction vector ${\vec{v}}_{v}^{F}\;$ is established by EX-HN3 and GV27 (Duiduan).

For the profile view, this model is applicable to the localization of TE21, SI19, and SI18 (Quanliao). The horizontal constraint $L_{h}^{P}\;$ selects $P_{h}^{P}$ as the profile reference point, and the direction vector ${\vec{v}}_{h}^{P}\;$ is determined by the ipsilateral inner and outer canthi. For the vertical constraint $L_{v}^{P}$, the anchor point is $P_{v}^{P}$, and the vertical direction vector ${\vec{v}}_{v}^{P}\;$ is defined as the orthogonal normal vector to ${\vec{v}}_{h}^{P}$ (i.e., ${\vec{v}}_{v}^{P}⊥{\vec{v}}_{h}^{P}$).

Ultimately, the target acupoint coordinates $P_{target}\;$ in any view can be obtained by solving for the intersection of the aforementioned geometric constraints. The generalized formula is:

(5) $$P_{target}=L(P_{h}^{k},{\vec{v}}_{h}^{k})\cap L(P_{v}^{k},{\vec{v}}_{v}^{k}),$$

where $L(P,\vec{v})\;$ represents a semi-infinite ray originating at point P and extending along the direction vector $\vec{v}$.

The fourth category is defined as the Directional Proportional Extension Method. Its core logic utilizes a keypoint as the origin and localizes the acupoint by extending a fixed proportional cun displacement along the horizontal or vertical direction. This model is optimal for acupoints with distinct nearby reference points, such as GB14 (extending 1 vertical cun upward from EX-HN4), BL1 (Jingming) (extending 0.1 vertical cun upward and 0.1 horizontal cun medially from the inner canthus), EX-HN5 (extending 1 horizontal cun outward from the midpoint between TE23 and GB1), and ST4 (Dicang) (extending 0.4 horizontal cun laterally from the mouth corner). Its vector extension formula is as follows:

(6) $$P_{target}=P_{ref}+k\cdot d_{unit}\cdot {\vec{n}}_{d}^{k},$$

where $P_{ref}\;$ is the anatomical reference origin; ${\vec{n}}_{d}^{k}\;$ is the unit vector indicating the extension direction; $d_{unit}\;$ is the unit cun length in the current view; and λ  is the proportional coefficient stipulated by TCM theory.

The fifth category is the Geometric Collinear Constraint Method. It applies to acupoints that exhibit collinear relationships with specific keypoints but lack explicitly defined cun measurements, such as ST1 (Chengqi) (directly below the pupil), LI20 (lateral to the lateral border of the nasal ala), CV24 (Chengjiang) (on the anterior median line), and TE23 (directly above GB1). Its core logic is to forcibly lock the acupoint coordinates onto a definitive linear trajectory:

(7) $$P_{target}=P_{ref}+t\cdot \vec{n},$$

where $\vec{n}\in \{{\vec{v}}_{h}^{k},{\vec{v}}_{v}^{k}\}\;$ is the dynamic directional basis constructed by the third method, and t is the displacement scalar parameter along this vector.

**Figure 2:**
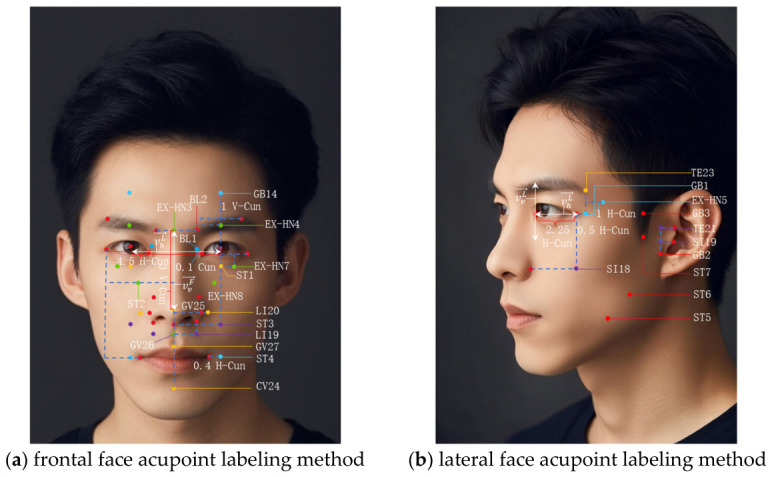
Schematic diagram of acupoint labeling.

Based on these five geometric constraint models, the specific localization criteria and calculation formulas for the 28 facial acupoints are detailed in Table 2 and Table 3. In the absence of tactile palpation, we achieved precise localization of ST2 (Sibai) and SI18 by combining strict adherence to national standards with methods from the literature [44,45]. Figure 2 visualizes the localization results. Scatter points in five colors (red, green, purple, blue, and yellow) represent the computational logic of the five methods. White bidirectional annotation lines indicate the measurement spans and vector directional baselines of the frontal and profile facial bone-proportional measurements. This system preserves the core of the TCM bone-proportional measurement method while adapting it to fine-grained coordinate regression under the thermal infrared modality. It supplies an automated and reproducibly verifiable annotation scheme for the 28 categories of facial acupoints and provides a theoretical foundation for subsequent indirect localization architectures.

**Figure 3:**
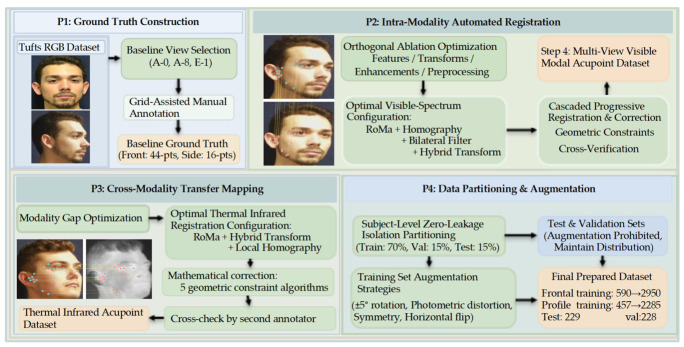
Stepwise data construction strategy.

To address annotation difficulties caused by low texture and ambiguous thermal boundaries, this study adopts a step-wise data construction strategy. As illustrated in Figure 3, the process starts from visible-light annotation and then proceeds through geometry-assisted consistency correction, manual review and local refinement, intra-modal registration, and cross-modal transfer to thermal images. In this workflow, geometric rules are used to support annotation consistency and efficiency, while the final reference coordinates are determined after image-based review and refinement.

First, to comprehensively support the geometric constraint algorithms described previously, a custom grid-assisted annotation tool was developed and integrated with the LabelMe software (v5.5.0) to manually annotate the TFD visible light subset (RGB-faces-128 × 128). The annotated content encompasses not only the target acupoints but also the auxiliary reference points requisite for localization. Specifically, the frontal face set contains a total of 44 keypoints (29 acupoints and 15 reference points), whereas the profile face set comprises 16 keypoints (11 acupoints and 5 reference points). Given the prohibitive cost and susceptibility to fatigue-induced errors associated with manual annotation across the entire dataset, this phase prioritized the annotation of standard profile faces (A-0 and A-8) and a subset of standard frontal faces (E-1). These subsets exhibit the most salient anatomical features and distinct spatial topological relationships, serving as the baseline ground truth for subsequent algorithmic expansion.

Upon acquiring the baseline ground truth, this study introduced image registration technology to automatically expand the coordinates to the remaining yaw angles of the subjects and performed cross-subject registration. To determine the final scheme, we initially evaluated a variety of traditional algorithms, including direct coordinate transfer, SIFT/ORB feature-based transformations, frequency-domain phase cross-correlation, B-spline non-rigid registration, and SimpleITK Demons. Experiments demonstrated that traditional methods exhibit low robustness when handling complex geometric deformations of human faces. In contrast, four cutting-edge deep learning-based models, namely RoMa [46], LoFTR [47], XoFTR, and SP_LG, demonstrated significant advantages in low-level feature extraction.

However, the feature points extracted by deep learning models typically only reflect the low-level textures or salient corner points of the image, which do not necessarily correspond precisely to the true anatomical locations of facial acupoints. Therefore, they cannot be used as acupoint coordinates, and a secondary mapping of the coordinates must be conducted in conjunction with spatial transformation mechanisms. To break through the accuracy bottleneck of cross-modal registration, this study designed a multivariate orthogonal ablation experiment. Specifically, at the feature matching end, the system evaluated the four aforementioned cutting-edge deep learning models. At the spatial transformation end, it compared eight methods including Homography, Affine, TPS, RBF, and their local variants. Simultaneously, it comprehensively cross-tested six feature enhancement strategies (including iterative, adaptive_ransac, and hybrid_transform) and four image preprocessing methods (including Bilateral and CLAHE). The results indicate that the cascade architecture comprising bilateral preprocessing, RoMa model feature extraction, and the hybrid transform strategy, specifically utilizing Homography for global coarse registration combined with RBF for local fine registration, achieved the optimal performance. This architecture reduced the Mean Error (ME) to 2.16 pixels, reduced the Normalized Mean Error (NME) to a global minimum of 1.68%, and demonstrated extremely high geometric fidelity in qualitative visual evaluations.

Furthermore, to address the severe perspective distortion induced by the large-angle deflection of profile faces, this study proposes an adjacent-angle-priority progressive registration strategy. This strategy utilizes the most precisely annotated standard profile faces (categories A-0 and A-8) as anchor frames, performing a smooth, progressive trajectory transition towards the frontal view. This approach effectively mitigates the non-linear distortion associated with drastic viewpoint transformations, further reducing the overall registration error by 0.66 to 1.71 pixels (yielding a maximum improvement of 10.17 pixels for a single trajectory).

Following the automated registration phase, a comprehensive expert review revealed residual algorithmic limitations in specific anatomical regions. For instance, the lower facial third of the frontal face is highly susceptible to facial expressions, where severe muscular deformations readily induce acupoint deviations. Concurrently, profile views are constrained by self-occlusion and projection angles, complicating pixel-level alignment for acupoints in the periauricular and outer canthus regions; as established previously, these regions lack symmetric mapping and inherently rely on global spatial reasoning. Additionally, anomalies such as occasional missing keypoints or erroneous annotations (e.g., left–right inversion of frontal acupoints and spatial misalignment of profile periauricular acupoints) were observed in isolated samples.

After automated registration, the five geometric constraint models were used for preliminary correction and consistency checking of non-rigid deformation and local feature deviations. The candidate coordinates were then cross-validated and refined by an independent annotator. Coordinates affected by expression, occlusion, weak thermal boundaries, large profile projection, or local registration errors were adjusted according to the image evidence and the standard acupoint atlas. The final reference coordinates are therefore manually confirmed annotations assisted by standard anatomical rules, not direct outputs of geometric formulas.

**Figure 4:**
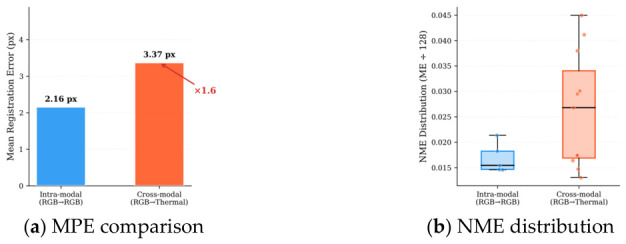
Statistical analysis chart of registration errors.

**Figure 5:**
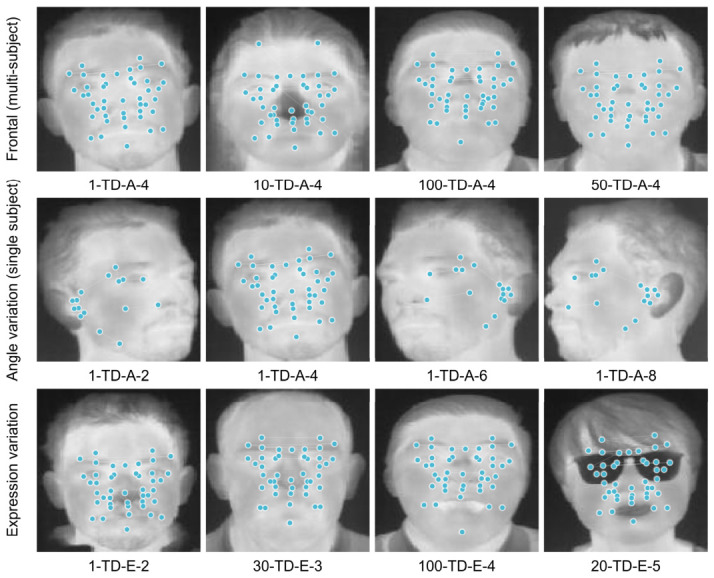
Examples of the thermal imaging acupoint localization dataset.

Finally, the aforementioned refined visible light dataset was registered to the TFD thermal imaging subset (Thermal-faces-128 × 128). To overcome the substantial physical modal gap between visible light and thermal infrared imagery, the registration module was re-optimized. Ultimately, a configuration comprising the RoMa model, the omission of preprocessing, hybrid feature enhancement, and Local Homography was identified as the optimal cross-modal scheme. While precisely anchoring feature regions, this scheme maximally preserves the relative topological structure of the facial acupoint clusters. Considering that the inherent error of cross-modal registration intrinsically remains higher than that of intra-modal transfer (as illustrated in Figure 4), a final phase of manual fine-tuning and careful screening was implemented post-automated mapping. Through this step-wise data construction pipeline, a high-precision, high-consistency thermal infrared facial acupoint localization dataset was successfully established (exemplified in Figure 5).

Given the limited scale of the TFD, we used a subject-independent partitioning and augmentation strategy to reduce overfitting and improve generalization. The training, validation, and testing sets were split by unique subject IDs at an approximate ratio of 70%, 15%, and 15%, respectively, rather than by random image-level assignment. All images from the same subject were assigned to only one subset, so the same person did not appear simultaneously in the training and testing sets. Data augmentation was applied only to the training set, while the validation and testing sets retained their original distributions for model selection and final evaluation.

Adhering to these partitioning principles, four image augmentation operations were applied to the training sets of both modalities. First, a bidirectional rotation of $\pm 5^{\circ }$ was introduced to account for postural tilts and non-strict alignments caused by physiological swaying during clinical acquisition, thereby enhancing spatial robustness. Second, photometric perturbations and Gaussian noise were applied to simulate the inherent thermal noise of uncooled infrared sensors and the brightness/contrast fluctuations induced by ambient temperature variations, improving cross-device generalization. Third, for frontal data, horizontal flipping coupled with label remapping was executed by leveraging facial physical symmetry; this explicitly compels the network to learn the geometric symmetric priors of the 45 frontal acupoints. Fourth, for profile data, given that the 16 lateral acupoints lack symmetric remapping and inherently rely on global spatial reasoning, horizontal flipping functions to simulate the contralateral perspective. This exposes the fine-grained regression network to diverse structural orientations without necessitating left-right independent distinction. Following a unified fivefold augmentation (comprising the original image and four enhancements), as detailed in Table 4, the frontal training set expanded from 590 to 2950 images. Applying an equivalent strategy, the profile training set expanded from 457 to 2285 images.

### 2.2. Proposed Method: Two-Stage Thermal Facial Acupoint Localization (T2FAL)

Traditional single-stage end-to-end keypoint detection architectures fail to maintain precision in thermal facial acupoint localization. The main difficulty arises from large physical and geometric differences between frontal and profile facial thermal distributions. In frontal views, the short infrared radiation path preserves high thermal signal fidelity and supports accurate temperature feature extraction. In large-angle profile views, longer radiation paths, perspective distortion, and temperature signal attenuation occur. In addition, profile angles cause severe spatial overlapping of anatomical structures in the 2D thermal projection. This produces clustered acupoint groups and self-occlusions that increase localization errors.

Coupling pose classification and fine-grained acupoint regression inside one monolithic network hinders the extraction of orientation-specific thermal features. This architectural coupling amplifies projection errors and reduces overall localization accuracy.

To address these constraints, this study develops the Two-Stage Thermal Facial Acupoint Localization (T2FAL) framework. The multi-pose localization task is divided into two modules. Stage 1 performs Thermal-Aware Face Detection, estimates the pose category (frontal or profile), and extracts aligned ROI crops. Stage 2 then performs pose-specific Progressive Acupoint Localization using coarse keypoint regression, feature-conditioned cascade refinement, and selective geometric constraints.

#### 2.2.1. Overall Architecture

**Figure 6:**
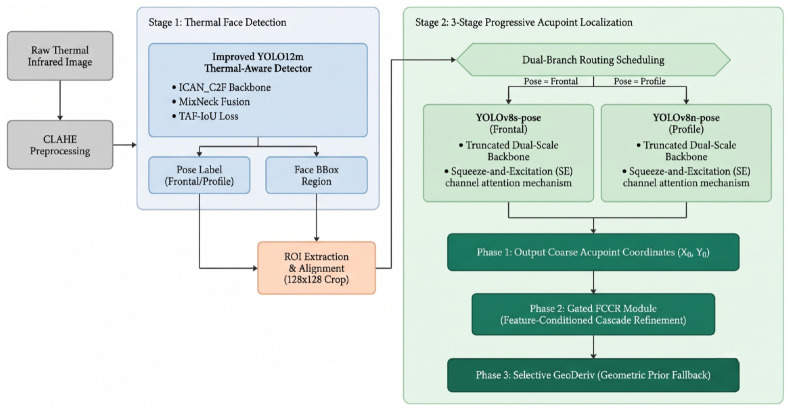
Two-stage thermal imaging facial acupoint detection flowchart.

As illustrated in Figure 6, the proposed T2FAL framework operates through a decoupled, sequential pipeline. Given a raw thermal infrared image, the system initially applies contrast enhancement preprocessing to attenuate thermal noise. The image is subsequently fed into Stage 1, where the improved YOLOv12m-based Thermal-Aware Face Detector simultaneously regresses the macroscopic facial bounding box and extracts the categorical pose prior (frontal or profile). Utilizing these spatial outputs, the face is cropped and aligned into a standardized 128 ×128 ROI. This aligned thermal ROI, guided by the extracted pose prior, subsequently activates the dual-branch routing scheduler in Stage 2. Contingent upon the facial orientation, the framework dynamically routes the image data to either the frontal or profile processing branch. Within the activated branch, the network executes a three-stage progressive localization mechanism: it initially predicts the coarse acupoint coordinates ($X_{0},Y_{0}$), subsequently applies Gated FCCR for dynamic sub-pixel optimization, and ultimately enforces a Selective Geometric (Selective GeoDeriv) module to provide a robust algorithmic fallback against severe topological occlusions. Through this meticulously decoupled paradigm, T2FAL effectively bridges macroscopic pose awareness with fine-grained acupoint precision.

#### 2.2.2. Stage 1: Thermal Face Detection and Pose Classification

**Figure 7:**
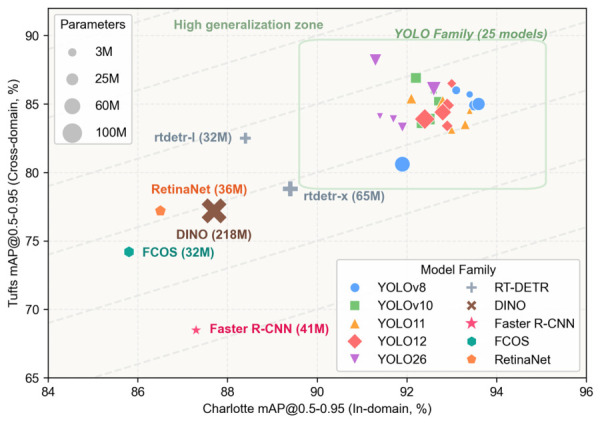
Baseline model architecture screening.

To select the optimal baseline detector, this study initially conducted a preliminary screening of 31 candidate models on the original CTF training set without data augmentation. The candidate array encompasses five generations of YOLO single-stage detectors (comprising 25 variant models ranging from YOLOv8 to YOLO26), end-to-end Transformer detectors (RT-DETR-l/x, DINO-SwinL), a classical two-stage detector (Faster R-CNN), and anchor-free detectors (FCOS, RetinaNet). All models were trained from scratch (Pre-trained = False, Epochs = 200), utilizing the mAP@0.5–0.95 on the CTF intra-domain validation set and the TFD cross-domain independent testing set as evaluation metrics. As illustrated in Figure 7, the non-YOLO models suffered from severe overfitting under limited infrared samples, confronting significant cross-domain performance attenuation and real-time processing bottlenecks.

For instance, models such as Faster R-CNN (42 M parameters) and DINO-SwinL (218 M) exhibited generalization gaps as substantial as 10.5 to 18.8 percentage points (pp), with inference latencies generally exceeding 25 ms. In contrast, based on the sum of intra-domain and cross-domain accuracies, the top 10 positions were exclusively secured by the YOLO series, with their cross-domain generalization gaps effectively suppressed between 3 and 9 pp. Accordingly, this study eliminated the non-YOLO models exhibiting weaker generalization capabilities, retaining the 25 YOLO variants for subsequent in-depth fine screening.

**Figure 8:**
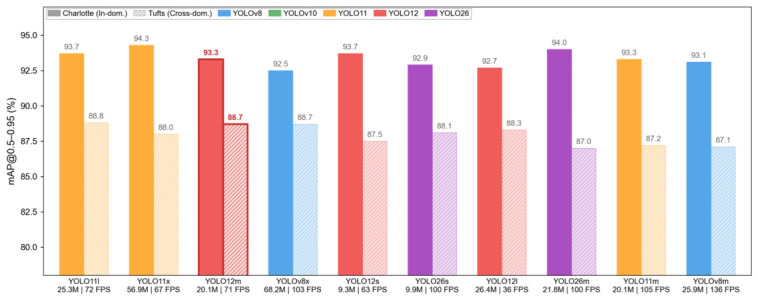
Evaluation of YOLO variants.

Subsequently, this study retrained the retained 25 YOLO models from scratch on the offline-augmented CTF_Aug dataset, conducting a multi-dimensional evaluation incorporating parameter counts and inference speeds. The results (Figure 8) indicate that YOLO12m aligns most optimally with the task requirements of this study. In the ranking based on the sum of mAP@0.5–0.95 from both datasets, YOLO12m (20.1 M parameters) ranked third with a score of 182.0, achieving intra-domain and cross-domain accuracies of 93.28% and 88.72%, respectively. Although its total score was marginally lower than the top two models, namely YOLO11L (182.55) and YOLO11x (182.26), YOLO12m showed strong parameter efficiency. Specifically, YOLO11L and YOLO11x possess parameter counts approximately 26% and 183% higher than YOLO12m, respectively, yet they achieved similar accuracy, outperforming YOLO12m by mere margins of 0.54 pp and 0.25 pp. Furthermore, YOLO12s, a lightweight variant within the same series, ranked fifth with only 9.3 M parameters, fully validating the high compatibility of the v12 architecture with thermal imaging data alongside its excellent architectural scalability. In summary, considering these factors together, this study selected YOLO12m as the optimal baseline detector for all subsequent architectural improvement experiments.

Although YOLO12m stands out in the comprehensive evaluation, it still faces two core bottlenecks when directly applied to thermal imaging scenarios. First, its cross-domain generalization capability remains limited, as evidenced by an accuracy drop of 4.56 pp on the independent testing set compared to the source domain. This attenuation exposes the risk that generic models are highly susceptible to overfitting on domain-specific textures present in the training set. Second, it lacks thermal awareness. Native attention mechanisms heavily rely on the structural and textural semantics of the targets, failing to explicitly model the specific signal distribution patterns inherent in infrared images—specifically, where target thermal radiation is intense with large channel variance, whereas background thermal radiation is weak with small channel variance. Consequently, this mismatch results in insufficient feature distinctiveness within the model.

**Figure 9:**
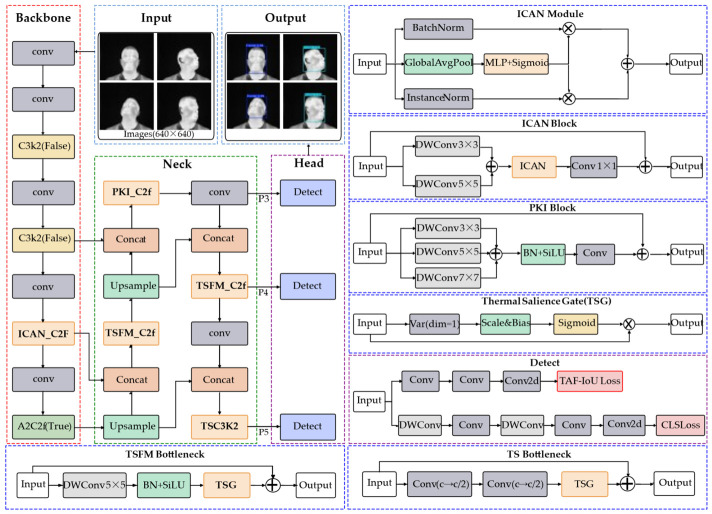
Architecture of the improved YOLO12m network and key module structures.

To overcome these bottlenecks, this study proposes a thermal-aware detection architecture customized specifically for infrared scenarios (the overall architecture is illustrated in Figure 9). This architecture reconstructs the baseline model across three dimensions: feature extraction, multi-scale fusion, and loss optimization. First, a three-stage thermal saliency gating (TSG) mechanism based on feature channel variance is designed and embedded as a unified plug-in within the backbone and feature fusion networks. This achieves targeted enhancement of thermal radiation signals alongside the suppression of cold background noise. Second, a heterogeneous neck fusion module (MixNeck) is constructed to maximally preserve the faint edge details of small targets while filtering redundant semantics through the differentiated processing of high-level and low-level pathways. Finally, a thermal adaptive focused loss (TAF-IoU) and a cosine temperature annealing strategy are adopted to achieve stage-adaptive bounding box regression optimization, thereby effectively tackling the challenge of hard examples induced by low infrared contrast and occlusions.

1. ICAN_C2f: Input-Conditional Adaptive Normalization Module in the Backbone Network

Thermal images are susceptible to significant domain shifts during acquisition due to fluctuations in camera parameters and ambient environments. The cross-domain generalization capability of standard Batch Normalization (BN) is limited. Conversely, Instance Normalization (IN), despite its ability to achieve domain invariance, tends to discard discriminative features. Inspired by the hybrid normalization concept of IBN-Net [48], this study designs an Input-Conditional Adaptive Normalization (ICAN) mechanism. This mechanism sequentially applies Global Average Pooling (GAP) and a two-layer Multilayer Perceptron (MLP) mapping—with a reduction ratio of r=16—to the input X. Following a Sigmoid activation, it generates channel-wise hybrid weights α=σ(MLP(GAP(X))). Ultimately, it outputs a dynamic interpolation between IN and BN:

(8) $$\hat{X}=\alpha ⊙\mathrm{IN}\left(X\right)+\left(1-\alpha \right)⊙\mathrm{BN}\left(X\right).$$

This mechanism adaptively adjusts α according to the degree of domain shift, achieving a dynamic balance between enhancing generalization and preserving discriminative features. Based on this, this study further constructs the ICAN_Bottleneck. It employs 3×3 and 5×5 Depthwise Separable Convolutions (DWConv) in parallel to extract multi-scale features, which are then processed by ICAN and a 1×1 convolutional projection before residual addition:

(9) $$Y=X+{\mathrm{Conv}}_{1\times 1}(\mathrm{ICAN}({\mathrm{DWConv}}_{3\times 3}(X)+{\mathrm{DWConv}}_{5\times 5}(X))).$$

Finally, the aforementioned Bottleneck is encapsulated within the Split-Concat topology of the baseline model’s standard C2f block to constitute ICAN_C2f. This newly designed module replaces the original A2C2f block exclusively in the P4 layer of the backbone network. The P4 layer is selected because its receptive field optimally matches the scale of medium-sized targets in infrared scenarios, and it is the location where cross-domain loss is most concentrated. In contrast, the P5 layer retains the original A2C2f block to maintain the indispensable global context modeling capability.

2. PKI_C2f: Multi-Scale Feature Capture Module in the Low-Level Fusion Pathway

In thermal images, target facial edges and contour signals carried by the low-level neck features (specifically at the P3 scale) are inherently faint and easily submerged by thermal noise. Consequently, a single-scale convolutional kernel struggles to achieve sufficient feature capture. To address this, this study introduces the PKIBlock proposed by PKINet [49] as the core building block and encapsulates it within the baseline’s C2f topology, thereby constructing the PKI_C2f module fully adapted to the YOLO12 architecture. The core mechanism of this module utilizes 3×3, 5×5, and 7×7 Depthwise Separable Convolutions to perform parallel feature extraction on the input X.

Following element-wise addition, the three-way multi-scale features sequentially undergo Batch Normalization (BN), SiLU activation, and a 1×1 convolutional projection, culminating in residual fusion with the input feature:

(10) $$Y=X+{Conv}_{1\times 1}\left(SiLU\left(BN\left(\sum_{k\in \{3,5,7\}}{DWConv}_{k\times k}\left(X\right)\right)\right)\right).$$

This multi-kernel parallel strategy enables the network to synchronously capture multi-scale structural information, thereby maximally preserving the faint thermal radiation details of the target—a capability essential for localizing minute facial acupoints. The rationale for deploying PKI_C2f within the low-level feature fusion pathway is that the P3 layer possesses the highest spatial resolution across the entire network. At this scale, target pixel representation is most sufficient, allowing the marginal utility of spatial feature extraction via multi-scale receptive fields to peak.

3. TSG and High-Level/Deep Thermal Gating Modules

The primary challenge confronting the high-level feature fusion pathway is that semantic features derived from deep layers are frequently entangled with background heat sources exhibiting temperatures similar to the human face. Consequently, thermal correlation filtering must be executed prior to fusion. This study observes that within the deep feature space, thermal radiation targets (e.g., human faces) exhibit high cross-channel variance due to the differential responses of various channels to thermal gradients. In contrast, background regions present very low variance owing to uniform thermal radiation.

Leveraging this physical prior, this study designs the Thermal Saliency Gate (TSG). Given an input tensor $X\in R^{B\times C\times H\times W}$, a spatial gate is generated by computing the variance along the channel dimension, which is subsequently scaled by learnable parameters α (initialized to 1) and β (initialized to 0):

(11) $$\hat{X}=X⊙\sigma \left(\alpha \cdot {\mathrm{Var}}_{c}(X)+\beta \right).$$

Containing only two scalar parameters, the TSG incurs negligible computational overhead, enabling its embedding across multiple levels of the neck network without compromising inference speed.

Building upon the TSG mechanism, this study constructs the TSFM_C2f module, which is deployed within the high-level fusion pathway of the neck. Its core unit, the TSFM_Bottleneck, employs a 5×5 DWConv to extract local spatial features. These features undergo BN and SiLU activation before being subjected to thermal filtering via the TSG:

(12) $$Y=X+{\mathrm{Conv}}_{1\times 1}(\mathrm{TSG}(\mathrm{SiLU}(\mathrm{BN}({\mathrm{DWConv}}_{5\times 5}(X))))).$$

As high-level features traverse the TSG, facial regions with salient thermal radiation are selectively enhanced, while background heat source noise characterized by flat variance is effectively suppressed. This endows the fused features with a substantially stronger capability for thermal target distinctiveness.

At the terminal end of the neck’s Path Aggregation Network (PAN) pathway (at the P5 scale), features are poised to enter the detection head. To perform a final round of thermal refinement at this critical juncture, this study deploys the TSC3k2 module. Specifically, the TSG is embedded at the output end of a standard Bottleneck (comprising two 3×3 convolutions with a channel scaling ratio of e=0.5), defined as:

(13) $$Y=X+\mathrm{TSG}({\mathrm{Conv}}_{3\times 3}({\mathrm{Conv}}_{3\times 3}(X))).$$

This configuration ensures that features entering the detection head undergo a final calibration for thermal saliency prior to classification and coordinate regression. TSC3k2 inherits the Split-Concat topology, and its parameter increment relative to the native C3k2 is virtually negligible.

Through these architectural designs, the TSG establishes a two-stage thermal gating strategy within the neck network—spanning the high-level pathway (TSFM_C2f, for thermal semantic filtering) and the deep terminal end (TSC3k2, for pre-prediction refinement). Working synergistically with PKI_C2f (for multi-kernel detail preservation) in the low-level pathway, these modules collectively constitute the comprehensive heterogeneous neck fusion strategy designated as MixNeck.

4. TAF-IoU:

Although the aforementioned architectural improvements improve feature extraction and fusion, bounding box regression in thermal scenarios still confronts inherent challenges. Thermal radiation gradients at target–background intersections frequently exhibit gradual transitions, which, coupled with self-occlusions, readily induce blurred boundaries. The standard CIoU [50] loss applies uniform weight gradients across all samples, rendering the effective optimization of such low-contrast hard examples highly challenging.

Consequently, this study proposes the Thermal-Adaptive Focusing IoU (TAF-IoU), synergizing the dynamic focusing mechanism of Wise-IoU [51] with a cosine temperature annealing schedule [52]. The focusing parameter β smoothly scales from 0 to $\beta_{\max}\;$(empirically set to 1.5 in this study) as the current training epoch tprogresses towards the total epochs T:

(14) $$\beta (t)=\frac{\beta_{max}}{2}\left(1-cos\left(\frac{\pi t}{T}\right)\right).$$

The regression loss calculation formula for TAF-IoU is formally defined as:

(15) $$L=(1-\mathrm{CIoU})\cdot e^{\left(1-\mathrm{CIoU})\cdot \beta (t\right)}\cdot w,$$

where w represents the intrinsic sample weight. The fundamental advantage of this stage-adaptive mechanism is its dynamic stabilization. During the early training stages when β≈0, TAF-IoU degenerates into the standard CIoU, guaranteeing stable model convergence. In the later training stages, as $\beta \to \beta_{\max}$, hard examples characterized by low IoU receive exponentially amplified gradient weights. This explicitly compels the network to perform fine-grained coordinate regression on ambiguous thermal boundaries. This progressive design effectively circumvents the adaptation imbalance associated with fixed weights across different training stages. Operating synergistically with the aforementioned architectural modifications, it establishes a complete algorithmic closed loop—coupling targeted feature-end enhancement with adaptive loss-end focusing.

#### 2.2.3. Stage 2: Pose-Guided Progressive Acupoint Localization

Building upon the Stage 1 pose detection, the second stage performs precise, fine-grained acupoint localization on the aligned facial regions. To select the optimal baseline, this study evaluates 21 keypoint models encompassing three major methodologies under a unified training protocol (128 × 128 resolution, Pretrained = False, 300 epochs). These candidates comprise 16 YOLO-Pose variants, four top-down heatmap-based models, and PFLD [53]. Given the significant generational gap observed in the localization performance of YOLOv12x, other YOLOv12 variants were excluded from this specific evaluation.

**Figure 10:**
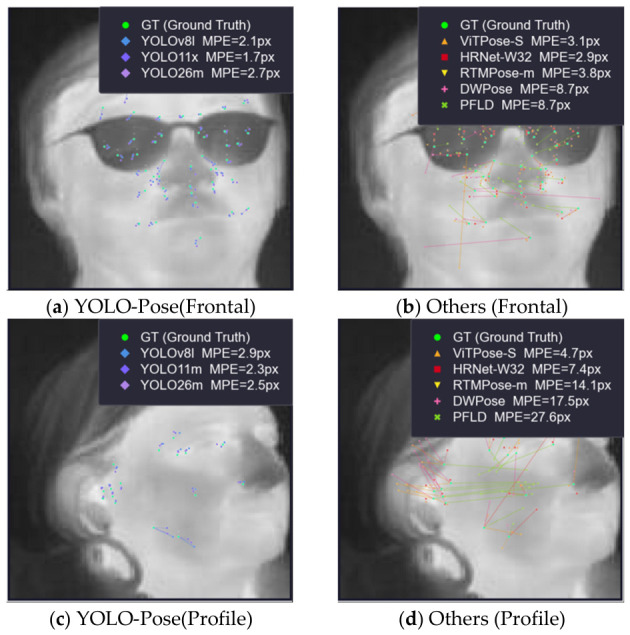
Qualitative comparison of keypoint predictions across detection paradigms on thermal facial images. Left column: YOLO-Pose (anchor-free regression); right column: Top-down heatmap-based and global regression models. Predictions are overlaid on the same test sample with ground truth (green circles) for reference.

The visualization results, illustrated in Figure 10, indicate that the predicted points of the three generations of YOLO-Pose (specifically v8, v11, and v26) closely fit the ground truth (GT). Their maximum frontal and profile deviations are within 3 pixels (px), maintaining a complete and topologically accurate structure. Conversely, the top-down series and PFLD exhibit widespread deviations and severe line intersections, completely losing their topological representation capabilities in profile scenarios. This degradation is attributed to two primary factors: computationally, heatmaps are highly susceptible to response aliasing for dense acupoints with a narrow spacing of 5 to 10 px under a low 128×128 input resolution; mathematically, the mixed distribution of left and right facial orientations in profile samples readily induces logical confusion during the spatial localization of identical acupoints by the model. Regarding computational efficiency, the inference latency of top-down models exceeds 22 ms, which is slower than the 5 to 7 ms achieved by the lightweight YOLO variants.

**Figure 11:**
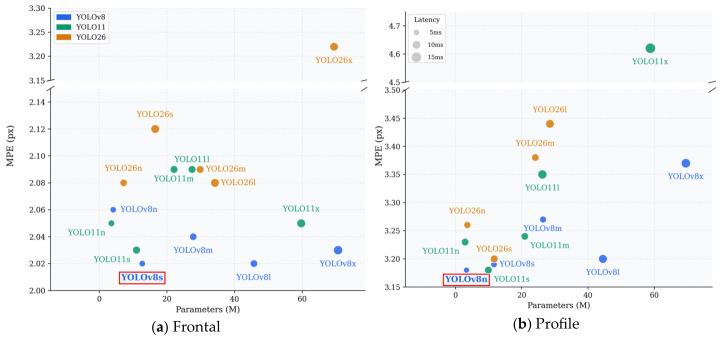
Performance and efficiency comparison of various YOLO architectures for thermal acupoint detection in (**a**) frontal and (**b**) profile views. Marker size represents inference latency. The red boxes indicate the base architectures selected for subsequent view-specific model development: YOLOv8s for frontal images and YOLOv8n for profile images.

Accordingly, after eliminating the severely degraded top-down models, PFLD, and YOLOv12x, this study focuses on the remaining 15 YOLO-Pose models evaluated in Figure 11. Quantitative results demonstrate that the frontal and profile Mean Pixel Errors (MPE) of these three YOLO generations stably converge into narrow intervals of 2.00 to 2.12 px and 3.18 to 3.45 px, respectively. Comprehensively considering accuracy, latency, and parameter efficiency, YOLOv8s-pose and YOLOv8n-pose are ultimately selected as the baseline architectures for subsequent Stage 2 optimization. Specifically, YOLOv8s-pose achieved a frontal MPE of 2.02 px and a latency of 5.80 ms utilizing 12.73 M parameters, while YOLOv8n-pose achieved a profile MPE of 3.18 px and a latency of 5.70 ms utilizing a mere 3.27 M parameters.

**Figure 12:**
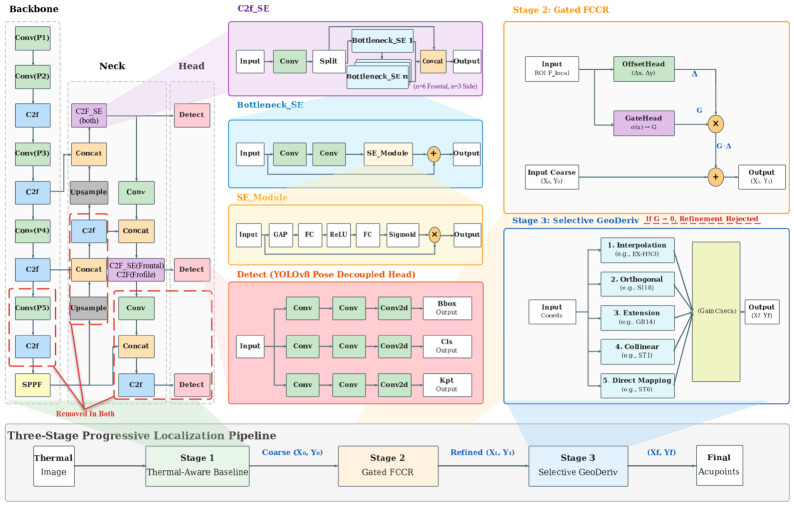
Detailed architecture of the Pose-Guided 3-Stage Progressive Acupoint Localization module.

This study proposes a three-stage progressive localization framework explicitly designed to address the deficiency of local texture characteristics in thermal imaging acupoint detection (as illustrated in Figure 12). Through a lightweight baseline network, gated feature refinement, and geometric prior derivation, this framework enhances localization accuracy while strictly constraining computational overhead, ultimately guaranteeing the reliability of facial acupoint detection. Under a unified input resolution of 128 ×128, the model is trained utilizing an AdamW optimizer and a cosine temperature annealing scheduler for 300 epochs, incorporating an early stopping mechanism with a patience of 51. The optimization is driven by a joint loss function comprising Object Keypoint Similarity (OKS) and CIoU.

To address the characteristics of thermal images, including the lack of high-frequency textures, low spatial resolution, and the susceptibility of deep features to dispersion effects, this study designs a minimalist backbone architecture. Deep features with large receptive fields in conventional object detection networks, such as the 32-fold downsampling layer of P5, not only struggle to extract effective topological semantics in thermal images but also easily introduce irrelevant background thermal noise. Therefore, this network thoroughly removes the conventional convolution and feature extraction modules (namely Conv and C2f) of the P5 stage from the architecture, retaining only the P3 and P4 layers to perform two-scale prediction. Notably, the Spatial Pyramid Pooling-Fast (SPPF) module at the tail of the original network is not directly discarded but is shifted forward to the terminal end of the P4 stage. Within the specific data forward propagation pathway, the C2f output of the P4 stage is truncated and directly connected to the SPPF. Subsequently, the multi-scale receptive field features refined by the SPPF serve as the highest-level semantic representation of the entire backbone network, being directly injected into the upsampling layers and bottom-up concatenation layers of the neck. While effectively truncating deep thermal noise and intensifying local spatial resolution, this design substantially reduces the parameter count of the backbone network by 64%.

To enhance the perception capability of the network regarding temperature gradients in acupoint neighborhoods, this study introduces the Squeeze-and-Excitation (SE) channel attention mechanism into the Bottleneck modules during the feature fusion stage. Furthermore, a differentiated attention deployment strategy is formulated to accommodate the variations in thermal feature distributions between frontal and profile faces. Because frontal acupoints are densely distributed and span widely, requiring high multi-scale spatial awareness, the model deploys attention modules with a higher depth (set to 6) at both the P3 and P4 levels. Conversely, profile acupoint localization relies more heavily on local high-frequency textures, where excessive aggregation of deep channel features easily induces model overfitting. Therefore, the specialized profile model precisely deploys shallow attention (set to 3) exclusively at the P3 node, while its P4 level retains the conventional residual convolutional structure. By adaptively adjusting channel weights, this mechanism strengthens the feature responses of local high-temperature sensitive regions and effectively suppresses noise interference. Ultimately, the extracted fused features are input into a decoupled pose head, which independently outputs the target bounding box, classification probability, and initial coarse coordinates $\left(X_{0}|Y_{0}\right)$ possessing preliminary topological robustness through three parallel branches.

To compensate for the localization deviations introduced by downsampling and coarse-resolution features in the initial stage, the second stage constructs a feature refinement module equipped with a safe isolation mechanism, designated as Gated Feature-Conditioned Cascade Refinement (Gated FCCR). Given the phenomenon of highly uneven thermal feature distributions across disparate facial regions, directly applying conventional local feature networks invariably leads to localization degradation in feature-sparse areas. This module performs local feature sampling on the ROI centered at $\left(X_{0}|Y_{0}\right)$ and constructs parallel gating branches. While predicting the spatial offsets (Δx|Δy), the model simultaneously generates a confidence gating weight G∈(0,1) via a Sigmoid activation function. The final refined coordinate updates are formulated as follows:

(16) $$\left(X_{1},Y_{1}\right)=\left(X_{0},Y_{0}\right)+G\cdot \left(\Delta x,\Delta y\right)$$

When a target keypoint resides in feature-sparse or topographically flat regions (e.g., the mandibular angle), the model adaptively outputs an extremely low correction confidence (G→0). This explicitly rejects unreliable fine-tuning operations, safely regressing to the macroscopic coarse-grained predictions derived from the first stage.

Even after network prediction and Gated FCCR refinement, some points may still show topological inconsistency under occlusion, facial hair, weak local texture, or local thermal disturbance. Selective GeoDeriv therefore reuses the five geometric constraint models as a rule-based post-processing step. These rules share the anatomical priors used during annotation assistance, but they do not generate the final reference coordinates. To limit conflicts with individual facial morphology, geometric derivation is applied only when it reduces validation-set error relative to the preceding network/FCCR output.

## 3. Results

This section evaluates the proposed T2FAL framework in two stages. We first assess the Thermal-Aware Face Detection module (Stage 1), including its robustness and cross-domain generalization. We then analyze the Progressive Acupoint Localization module (Stage 2) and quantify the contribution of each refinement component.

### 3.1. Evaluation of Thermal Face Detection

To verify the independent contributions and synergistic effects of each architectural modification, this section designs two sets of ablation experiments under strictly controlled variables: the independent contribution of single modules and the synergistic effect of module combinations. All experiments employ identical training configurations (the CTF_Aug dataset, random seed = 0, 200 epochs, 640 × 640 input resolution, and batch size = 16) and a consistent hardware environment (a single NVIDIA RTX 4090 GPU). Evaluations are conducted on both the CTF (intra-domain) and TFD (cross-domain) independent testing sets.

To quantify the independent contribution of each module, this study utilizes the baseline YOLOv12m and replaces only one architectural component at a time, keeping the remainder unchanged. The experimental results, detailed in Table 5, demonstrate that the four independent modules effectively enhance the baseline model’s performance across multiple dimensions. Introducing the ICAN_C2f backbone not only reduces the parameter count by 8.6%, but its adaptive normalization mechanism also exhibits a significant advantage in feature preservation, elevating the Cm95 metric to a peak of 94.31 (the highest among all single modules). Furthermore, TSFM_C2f substantially strengthens the model’s cross-domain generalization capability, achieving the highest cross-domain metric Tm95 of 91.46. This explicitly validates the effectiveness of the thermal saliency gating (TSG) mechanism in suppressing cross-domain background noise.

On the other hand, the PKI_C2f and TSC3k2 modules exhibit balanced optimization effects across the holistic metrics. The PKI_C2f neck, employing a multi-kernel parallel strategy, demonstrates a significant capability for missed detection suppression (evidenced by high Recall). The TSC3k2 module, located in the deep terminal layers, achieves the highest intra-domain basic localization accuracy (Cm51) of 99.50% without inflating the parameter count, simultaneously realizing synchronous improvements in precision and recall during cross-domain testing. These single-factor experimental results fully validate the efficacy of each independent module, laying a solid quantitative foundation for the complementary feature fusion in the subsequent heterogeneous network evaluation.

The aforementioned experiments effectively verify the independent value of each module; however, the synergistic working mechanism among these modules must be considered for actual deployment. To this end, this study designs progressive combinatorial experiments to explore feature fusion effects, with the results detailed in Table 6.

Although ICAN_C2f and TSFM_C2f achieved optimal values on the intra-domain and cross-domain mAP51–95 metrics respectively during independent testing, both metrics exhibited a decline following their direct combination. This attenuation indicates the existence of a specific feature competition relationship between them. Since this competition occurs during the feature transmission stage within the neck, this study initially attempted to perform secondary refinement on the pre-prediction features by superimposing TSC3k2 in the deep terminal layers without altering the core neck topology. The results demonstrate that the introduction of TSC3k2 recovered the cross-domain recall (TR) to 97.74 and elevated Tm95 to 89.52; nevertheless, the intra-domain Cm95 stagnated at 93.41. This phenomenon indicates that executing feature refinement exclusively within the deep layers is insufficient to resolve the competition issue, necessitating a fundamental solution originating from the neck structure itself.

Therefore, this study further incorporates PKI_C2f—forming the comprehensive MixNeck heterogeneous architecture—to preserve spatial edge information within the low-level pathways. Under the configuration comprising ICAN_C2f and MixNeck, both intra-domain and cross-domain metrics improved synchronously. The comprehensive metric Sum reached 769.86, with all 10 evaluated metrics outperforming the baseline. To further enhance bounding box regression accuracy specifically targeting hard examples, the TAF-IoU loss function was ultimately introduced. The complete, finalized model improves cross-domain localization accuracy and the comprehensive score while concurrently achieving substantial parameter reduction and inference acceleration. This outcome supports the efficacy of the joint optimization strategy coupling heterogeneous feature fusion with adaptive loss scaling.

Synthesizing the aforementioned ablation experiment results, each improved module assumes a distinct functional division within the overall architecture. The ICAN_C2f backbone provides the feature foundation for intra-domain high-precision localization via adaptive normalization. The MixNeck heterogeneous neck resolves the intra-domain and cross-domain feature competition problem through a hierarchical feature processing strategy. Ultimately, the TAF-IoU loss function further enhances bounding box regression accuracy specifically targeting hard examples. Under the conditions of an 11.4% reduction in parameter count and a 19.9% increase in inference speed, all 10 evaluation metrics of the final full model outperform the baseline. The comprehensive score elevated impressively from 748.94 to 772.26, thereby fully validating the effectiveness and necessity of the holistic architectural design.

**Figure 13:**
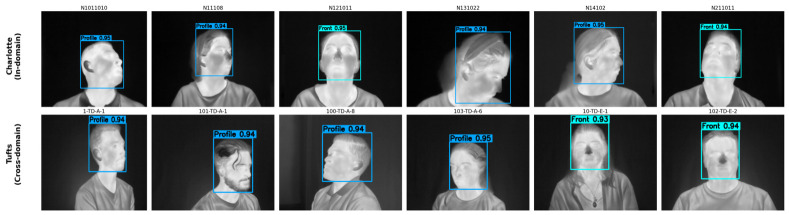
Detection results of the improved model.

To intuitively demonstrate the detection performance of the finalized full model, Figure 13 presents the visualization results on both the intra-domain CTF testing set and the cross-domain TFD testing set. The selected samples encompass infrared facial images across various subjects, head orientations, and diverse imaging conditions.

Within the intra-domain CTF testing set, the model correctly localizes and classifies multiple poses. The detection confidences are concentrated within the 0.94 to 0.95 interval, and the bounding boxes tightly fit the facial boundaries. Regarding the cross-domain TFD testing set, despite significant discrepancies in imaging equipment, image resolution, and subject demographics compared to the source training set, the model still achieves stable, robust detection for both frontal and profile faces across various spatial orientations, with confidences ranging consistently from 0.93 to 0.95.

### 3.2. Performance of Progressive Acupoint Localization

To objectively evaluate the performance of the proposed three-stage progressive localization framework in the thermal imaging acupoint detection task, this study conducted comparative experiments with the baseline model, YOLOv8-Pose, under strictly controlled variables. All models were trained from scratch under identical hardware and data augmentation strategies (300 epochs and patience = 51). The primary evaluation metrics encompass localization accuracy metrics, including MPE to measure absolute physical deviation, NME to evaluate scale invariance, and PCK@0.2 to reflect the macroscopic hit rate, alongside efficiency metrics, including parameter count (Params) and inference latency (Latency), to evaluate the potential for lightweight deployment on medical-grade terminals.

The experimental results are detailed in Table 7 and Table 8. In both frontal and profile thermal face testing, the improved architecture outperforms the baseline model. Specifically, in frontal face testing, the improved model achieves significant lightweighting, with the parameter count sharply reduced by 63.6% and single-frame inference latency decreased by 21.2%. While reducing computational overhead, localization accuracy achieves contrarian optimization. MPE drops to 2.013 px, PCK@0.2 increases to 40.51%, and both mAP@50–95 and NME exhibit superior robustness. Regarding profile acupoints characterized by more complex and asymmetric feature distributions, the improved model performs well. It not only comprehensively elevates accuracy metrics but also extremely compresses the parameter count to 1.10 M and reduces inference latency to 5.96 ms (approximately 168 FPS). This fully corroborates the exceptional potential of this framework to achieve low-latency and real-time localization on resource-constrained edge computing devices.

To verify the independent contributions and cross-domain generalization capabilities of each component within the framework, this study conducted ablation experiments (Table 9 and Table 10). The experimental data clearly present the cumulative gains of the progressively superimposed optimization strategies. Analyzing the ablation results from both datasets reveals several findings. First, removing the P5 layer cuts the parameter count by over 63% without causing accuracy degradation, corroborating the necessity of streamlining the deep receptive field to filter out noise. Second, the channel attention mechanism introduced to address the thermal distribution differences across domains successfully suppresses background interference, reducing the frontal MPE to 2.013 px. Building upon this, the Gated FCCR mechanism achieves robust refinement of macroscopic localization with minimal computational overhead (less than 0.1 M), further depressing the frontal extreme error to 1.996 px by relying on the dynamic gating mechanism. Finally, the introduced Selective GeoDeriv mechanism, serving as a spatial mathematical prior, pushes the frontal and profile MPE down to 1.986 and 3.109 px respectively under the premise of zero-parameter and zero-latency increments. Furthermore, it comprehensively elevates the macroscopic hit rate against occlusions, effectively enhancing the stability and safety of topological structures during model inference.

We further analyzed the landmark-wise effect of Selective GeoDeriv relative to Gated FCCR. For each landmark, the mean delta was defined as the error after full geometric correction minus the error after Gated FCCR. Negative values therefore indicate error reduction, whereas positive values indicate error increase.

**Figure 14:**
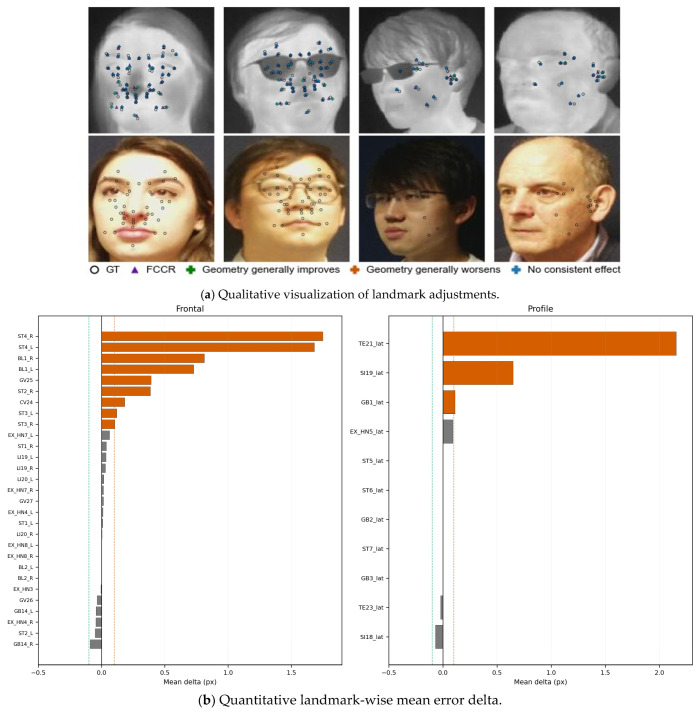
Landmark-wise effect of Selective GeoDeriv relative to Gated FCCR. (**a**) Qualitative visualization of representative examples showing Ground Truth (GT), FCCR output, and GeoDeriv-adjusted points. The color of each GeoDeriv point reflects the global landmark-level statistics rather than a single-image threshold. (**b**) Quantitative landmark-wise mean error delta, calculated as the full GeoDeriv error minus the Gated FCCR error. Negative values indicate error reduction (geometry improves), positive values indicate error increase (geometry worsens), and near-zero values indicate no consistent effect. For both subfigures, the marker and bar colors denote the corresponding statistical trend: green indicates improvement, orange indicates worsening, and gray indicates a near-neutral effect.

Figure 14 shows that the effect of GeoDeriv varies across landmarks. In frontal views, several eyebrow-, infraorbital-, nasal-root-, and midline-related regions showed small average improvements. In contrast, some mouth-corner-, inner-canthus-, and nasal-tip-related regions did not consistently benefit, probably because of lip motion, exhaled heat, eyeglass interference, nasal protrusion, and mild asymmetry. In profile views, the improvement was more limited, as periauricular and temporal landmarks are sensitive to projection changes, individual ear morphology, hair occlusion, and weak thermal boundaries.

### 3.3. External Qualitative Checks

**Figure 15:**
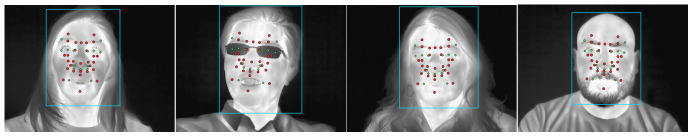
Qualitative external and challenging-sample analysis on SF-TL54 thermal facial images. The trained Stage 2 model was directly applied without retraining or dataset-specific fine-tuning. These examples illustrate structured localization under external acquisition conditions and common challenging factors such as eyeglasses, facial hair, hair occlusion, expression variation, and local weak thermal boundaries.

We also tested the trained Stage 2 model on SF-TL54 thermal facial images without retraining or dataset-specific fine-tuning. SF-TL54 differs from the present dataset in imaging device, face scale, head pose, local contrast, and common occlusions. As shown in Figure 15, the model still produced structured facial acupoint distributions on these external images. The same examples also illustrate practical challenges: eyeglasses and facial hair did not completely disrupt the overall frontal structure, but mouth-region landmarks were more sensitive to expression, lip state, and exhaled heat; profile and periauricular landmarks were more affected by head rotation, hair occlusion, and weak thermal boundaries. Because SF-TL54 does not provide expert acupoint GT, this analysis is qualitative rather than a quantitative external validation.

## 4. Discussion

The experimental results of the proposed T2FAL framework address the three challenges of infrared thermal imaging facial acupoint localization identified at the outset of this study: texture scarcity, dataset deficiency, and multi-pose coordinate offset. With an mAP@50–95 of 73.19% (NME = 2.68%) on frontal views and 86.92% (NME = 4.895%) on profile views, T2FAL outperforms the single-stage baselines evaluated under the same protocol. These results can be attributed to three design choices: (1) explicitly decoupling pose estimation from keypoint regression prevents error propagation across different radiation paths and projection geometries; (2) the heterogeneous MixNeck architecture aligns feature extraction with physical thermal radiation priors, suppressing background noise while preserving faint topological details; and (3) selective geometric constraints rooted in national standards (GB/T) and the bone-proportional measurement system compensate for residual projection distortions that purely data-driven approaches cannot eliminate.

In the context of recent acupoint-localization methods, YOLOv8-ACU integrates ECA and achieves real-time facial acupoint detection on visible-light images, while AIR-Net employs atlas-based registration for hand dorsum acupoints under varied skin tones. Both methods were developed and evaluated on RGB data; their performance under thermal infrared conditions has not been reported. Compared with these RGB-modality approaches, T2FAL operates directly on thermal images and incorporates explicit pose priors to handle the frontal/profile projection difference—a scenario where single-view architectures tend to produce coordinate overlap among periauricular acupoints. Earlier infrared-specific studies, such as SSD + Dlib pipelines for forehead acupoint temperature measurement, addressed only a limited number of frontally visible acupoints without geometric constraints. The present framework extends the localization scope to 28 acupoint categories across both views and provides a digitized cun measurement system compliant with GB/T 12346-2021 and GB/T 40997-2021 to support annotation reproducibility.

Taken together, these results support the feasibility of standardized facial acupoint localization in thermal infrared images. By translating bone-proportional measurement rules into computable geometric constraints, T2FAL provides anatomically aligned coordinates for subsequent region-based thermal analysis. Such alignment is useful because thermal features are more comparable when they are extracted from consistent anatomical regions. The present study does not include disease labels, syndrome labels, or prospective clinical outcomes; accordingly, screening or diagnostic performance is not inferred from the current experiments.

Several limitations should be noted. First, although the current dataset is a relatively large thermal facial acupoint dataset, it is still modest in scale and mainly derived from controlled laboratory conditions. Severe facial asymmetry, craniofacial abnormality, obvious scars, or postoperative structural changes were not fully represented and should be considered potential out-of-distribution scenarios. Second, thermal facial images can be influenced by environmental temperature and humidity, adaptation time, recent physical activity, cosmetics, local inflammation, respiratory airflow, sweating, skin surface moisture, and camera resolution or calibration. Hair, hats, eyeglasses, facial hair, expression, local occlusion, and image blur may further increase localization uncertainty, especially in profile views and low-contrast regions. Finally, although the landmark-wise analysis shows that GeoDeriv does not uniformly improve all landmarks, complete external anatomical validation will require an independent dataset or multi-expert consensus annotations.

Future work should expand the dataset to larger multi-center and multi-modal cohorts and use stricter thermal acquisition protocols with recorded environmental and subject-state variables. Independent clinical-expert comparison is also needed. Because direct facial acupoint annotation on thermal infrared images alone is uncertain, future expert protocols should combine visible-light images, thermal images, standard acupoint atlases, and necessary anatomical references under blinded conditions. Model predictions can then be compared with expert consensus coordinates and inter-expert variability. On this basis, standardized acupoint coordinates may support regional thermal-feature extraction and subsequent clinical association studies using explicit disease or syndrome labels.

## 5. Conclusions

This study presents T2FAL, a two-stage cascaded regression framework explicitly designed to resolve the inherent challenges of texture scarcity, dataset deficiency, and perspective-induced coordinate overlap in thermal facial acupoint localization. By decoupling macroscopic face detection from fine-grained acupoint regression, the proposed framework provides a highly stable and robust alternative to monolithic networks. Experimental evaluations demonstrate highly competitive performance. In the first stage, the model achieves a comprehensive score of 772.26 at an inference speed of 87.3 FPS. In the second stage, it reduces the Mean Pixel Error (MPE) to 1.986 pixels (mAP@51–95 of 73.19%) for frontal views and 3.109 pixels (mAP@51–95 of 86.92%) for profile views.

The principal contributions of this work are twofold. First, it establishes an integrated thermal facial acupoint dataset featuring GB/T-standardized digitized body-cun annotations alongside five geometric constraint models. Second, it develops the complete T2FAL framework, a two-stage architecture comprising Thermal-Aware Face Detection and Progressive Acupoint Localization with lightweight feature extraction, channel attention, Gated FCCR, and Selective GeoDeriv refinement. Overall, T2FAL provides a standardized localization framework for facial acupoints in thermal infrared images and a technical basis for future thermal-zone analysis; its clinical diagnostic value remains to be validated in disease- or syndrome-specific studies.

## Figures and Tables

**Table 1:** Data partitioning and distribution for the first-stage thermal imaging frontal and profile face detection task.

Dataset	Data Characteristics	Train (Orig./Aug.) [F/P]	Val [F/P]	Test [F/P]	Total Samples (Orig./Aug.) [F/P]
TFD	Discrete angles	1052/4208 [2396/1812]	225 [144/81]	227 [106/121]	1504/4660 [2646/2014]
CTF	High-DoF poses	721/2884 [1060/1824]	154 [65/89]	155 [55/100]	1030/3193 [1180/2013]

**Table 2:** Summary of geometric modeling and localization formulas for frontal facial acupoints.

Category	Acupoint (Code)	Geometric Localization Formula	Standard Reference	Standard Position Description
Direct Mapping	Cuanzhu (BL2)	$P_{BL2}=P_{brow\_in}$	GB/T 12346-2021 5.7.2	Depression at medial eyebrow end, on supraorbital notch.
	Shangyingxiang (EX-HN8)	$P_{EX-HN8}=P_{naso\_top}$	GB/T 40997-2021 7.1.7	Upper end of nasolabial groove.
Decoupled Interpolation	Yintang (EX-HN3)	$x=\frac{x_{BL2\_L}+x_{BL2\_R}}{2}$ $y=\frac{y_{BL2\_L}+y_{BL2\_R}}{2}$	GB/T 12346-2021 5.13.25	Midpoint between bilateral BL2.
	Yuyao (EX-HN4)	$x=x_{pupil}$ $y=y_{BL2}+0.5\left(y_{peak}-y_{BL2}\right)$	GB/T 40997-2021 7.1.3	Center of eyebrow, directly above pupil.
	Qiuhou (EX-HN7)	$x=x_{LC}+0.25\left(x_{IC}-x_{LC}\right)$ $y=y_{ST1}$	GB/T 40997-2021 7.1.6	Between eyeball and infraorbital margin (lateral 1/4).
	Sibai (ST2)	$x_{AM}=\frac{x_{BL2\_L}+x_{BL2\_R}}{2}$ $x=x_{AM}+\left(x_{LC}-x_{AM}\right)\times \frac{27.2}{41.3}$ $y=y_{LC}+\left(y_{mouth}-y_{LC}\right)\times \frac{25.1}{71.3}$	GB/T 12346-2021 5.3.2; Literature [44]	0.66 from nasal midline to LC; 0.35 from LC to mouth corner.
	Shuigou (GV26)	$x=\frac{x_{alar\_L}+x_{alar\_R}}{2}$ $y=y_{sn}+\frac{1}{3}\left(y_{GV27}-y_{sn}\right)$	GB/T 12346-2021 5.13.27	Junction of upper and middle 1/3 of philtrum.
Dual-Line Orthogonal	Juliao (ST3)	$L\left(P_{sn},\vec{v_{lat}^{F}}\right)\cap L\left(P_{pupil},\vec{v_{down}^{F}}\right)$	GB/T 12346-2021 5.3.3	Level with lower ala nasi, directly below pupil.
	Kouheliao (LI19)	$L\left(P_{GV26},\vec{v_{lat}^{F}}\right)\cap L\left(P_{alar\_mid},\vec{v_{down}^{F}}\right)$	GB/T 12346-2021 5.2.19	Level with GV26, directly below lateral nostril.
Directional Extension	Yangbai (GB14)	$P_{EX-HN4}+1.0\cdot d_{unit}\cdot \vec{n_{up}^{F}}$	GB/T 12346-2021 5.11.14	1 cun above eyebrow, directly above pupil.
	Jingming (BL1)	$P_{IC}+0.1\cdot d_{unit}\cdot \left(\vec{n_{up}^{F}}+\vec{n_{med}^{F}}\right)$	WHO Standard	0.1 cun superior-medial to inner canthus.
	Dicang (ST4)	$P_{mouth}+0.4\cdot d_{unit}\cdot \vec{n_{lat}^{F}}$	GB/T 12346-2021 5.3.4	0.4 cun lateral to mouth corner.
Collinear Constraint	Chengqi (ST1)	$P_{pupil}+t\cdot \vec{n_{down}^{F}}$	GB/T 12346-2021 5.3.1	Between eyeball and infraorbital margin, directly below pupil.
	Yingxiang (LI20)	$P_{alar\_mid}+t\cdot \vec{n_{lat}^{F}}$	GB/T 12346-2021 5.2.20	Nasolabial sulcus, level with lateral ala nasi midpoint.
	Suliao (GV25)	$\frac{P_{alar\_L}+P_{alar\_R}}{2}+t\cdot \vec{n_{up}^{F}}$	GB/T 12346-2021 5.13.26	Nose tip.
	Duiduan (GV27)	$\frac{P_{mouth\_L}+P_{mouth\_R}}{2}+t\cdot \vec{n_{up}^{F}}$	GB/T 12346-2021 GV27	Upper lip midline, at skin-mucosa junction.
	Chengjiang (CV24)	$\frac{P_{mouth\_L}+P_{mouth\_R}}{2}+t\cdot \vec{n_{down}^{F}}$	GB/T 12346-2021 5.14.24	Mentolabial sulcus depression.

pupil (pupil center), peak (highest point of the eyebrow), brow_in (medial end of the eyebrow), LC/IC (lateral/inner canthus), sn (columella), alar_L/alar_R (left/right lateral border of ala nasi), alar_mid (midpoint of the lateral border of ala nasi), naso_top (upper end of the nasolabial groove), mouth_L/mouth_R/mouth (left/right/unilateral corner of the mouth), AM (midpoint of the line connecting the left and right ala nasi).

**Table 3:** Summary of geometric modeling and localization formulas for lateral facial acupoints.

Category	Acupoint (Code)	Geometric Localization Formula	Standard Reference	Standard Position Description
Direct Mapping	Shangguan (GB3)	$P_{GB3}=P_{zygoma\_up}$	GB/T 12346-2021 5.11.3	Upper border of zygomatic arch.
	Xiaguan (ST7)	$P_{ST7}=P_{zygoma\_low}$	GB/T 12346-2021 5.3.7	Lower border of zygomatic arch.
	Tinghui (GB2)	$P_{GB2}=P_{tragus\_pre}$	GB/T 12346-2021 5.11.2	Anterior to intertragic notch.
	Jiache (ST6)	$P_{ST6}=P_{masseter}$	GB/T 12346-2021 5.3.6	1 finger-width anterior-superior to mandibular angle.
	Daying (ST5)	$P_{ST5}=P_{artery}$	GB/T 12346-2021 5.3.5	Anterior to mandibular angle, on anterior masseter border.
Dual-Line Orthogonal	Ermen (TE21)	$L\left(P_{sup\_trag},\vec{v_{med}^{L}}\right)\cap L\left(P_{GB2},\vec{v_{up}^{L}}\right)$	GB/T 12346-2021 5.10.21	Depression anterior to supratragic notch.
	Tinggong (SI19)	$L\left(P_{trag\_mid},\vec{v_{med}^{L}}\right)\cap L\left(P_{GB2},\vec{v_{up}^{L}}\right)$	GB/T 12346-2021 5.6.19	Between tragus and mandibular joint.
	Quanliao (SI18)	$L\left(P_{alar\_mid},\vec{v_{lat}^{L}}\right)\cap L\left(P_{LC},\vec{v_{down}^{L}}\right)$	Literature [45]	Intersection of outer canthus vertical line and ala nasi horizontal line.
Directional Extension	Tongziliao (GB1)	$P_{LC}+0.5\cdot d_{unit}\cdot \vec{n_{lat}^{L}}$	GB/T 12346-2021 5.11.1	0.5 cun lateral to outer canthus.
	Taiyang (EX-HN5)	$\frac{P_{TE23}+P_{GB1}}{2}+1.0\cdot d_{unit}\cdot \vec{n_{lat}^{L}}$	GB/T 12346-2021 7.1.4	1 cun lateral to midpoint between TE23 and GB1.
Collinear Constraint	Sizhukong (TE23)	$P_{TE23}=P_{GB1}+t\cdot \vec{n_{up}^{L}}$	GB/T 12346-2021 5.10.23	Lateral end of the eyebrow, directly above GB1.

brow_out (lateral end of the eyebrow), LC (lateral canthus), alar_mid (midpoint of the lateral border of ala nasi); lateral skeletal and muscular landmarks include: zygoma_up/zygoma_low (upper/lower border of the zygomatic arch), tragus_pre (anterior to the intertragic notch), sup_trag (supratragic notch), trag_mid (middle of the tragus), masseter (prominence of the masseter muscle), artery (facial artery notch).

**Table 4:** Division of the acupoint localization dataset.

Dataset Category	Number of Keypoints	Training Set (Orig./Aug.)	Validation Set	Test Set
Frontal data	44	590/2950	125	134
Profile data	16	457/2285	104	94

**Table 5:** Ablation experiment results of the single-factor independent contributions for each improved module on the Charlotte and Tufts testing sets.

Config	Par	FPS	CP	CR	Cm50	Cm95	TP	TR	Tm50	Tm95	Sum	Pass
Baseline	20.14	72.8	98.22	97.69	99.35	93.41	86.39	90.29	95.46	88.13	748.94	—
+ ICAN_C2f	**18.41**	**77.5**	97.50	**99.51**	**99.47**	**94.31**	**91.76**	**92.30**	**96.84**	**88.90**	**760.59**	9/10
+ TSFM_C2f	**19.56**	**81.1**	**98.34**	**99.23**	**99.40**	**93.53**	**95.18**	**96.54**	**98.76**	**91.45**	**772.43**	10/10
+ PKI_C2f	**19.60**	**77.5**	96.03	**99.82**	**99.39**	**93.86**	**89.10**	**93.77**	**96.45**	**88.92**	**757.34**	9/10
+ TSC3k2	20.14	**74.0**	**98.43**	**98.53**	**99.49**	**93.72**	**91.17**	**94.77**	**97.30**	**88.76**	**762.17**	9/10

Bold values indicate improvements over the baseline, whereas bold and underlined values indicate the best result in each column. Lower values are better for Par, whereas higher values are better for FPS and the accuracy metrics. “C” and “T” denote results on the intra-domain CTF and cross-domain TFD test sets, respectively. “Sum” is the sum of the eight accuracy metrics. “Pass” indicates the number of metrics that outperform the baseline among the ten evaluated metrics, including Par and FPS.

**Table 6:** Ablation experiment results of the progressive synergistic effects for module combinations.

Config	Par	FPS	CP	CR	Cm50	Cm95	TP	TR	Tm50	Tm95	Sum	Pass
Baseline	20.14	72.8	98.22	97.69	99.35	93.41	86.39	90.29	95.46	88.13	748.94	—
+ ICAN_C2f	**18.41**	**77.5**	97.50	**99.51**	**99.47**	**94.31**	**91.76**	**92.30**	**96.84**	**88.90**	**760.59**	9/10
+ TSFM_C2f	**17.80**	**80.9**	96.02	**99.70**	**99.44**	**93.95**	**91.84**	**90.55**	**96.89**	**88.36**	**756.75**	9/10
+ ICAN_C2f + TSFM_C2f + TSC3k2	**17.83**	**90.2**	**98.99**	**98.54**	**99.48**	93.40	**87.65**	**97.74**	**97.03**	**89.51**	**762.34**	9/10
+ ICAN_C2f + MixNeck	**17.84**	**89.4**	**98.38**	**99.90**	**99.49**	**93.70**	**94.59**	**95.11**	**98.41**	**90.28**	**769.86**	10/10
Full Model	**17.84**	**87.3**	**98.53**	**98.62**	**99.47**	**93.72**	**95.35**	**97.18**	**98.95**	**90.44**	**772.26**	10/10

Bold values indicate results that outperform the baseline, whereas bold and underlined values indicate the best result in each column, including ties. Lower values are better for Par, whereas higher values are better for FPS, the accuracy metrics, Sum, and Pass. “C” and “T” denote results on the intra-domain CTF and cross-domain TFD test sets, respectively. The Pass column reports the number of indicators outperforming the baseline among the ten evaluated indicators, comprising eight accuracy metrics, Par, and FPS. MixNeck consists of TSFM_C2f, PKI_C2f, and TSC3k2.

**Table 7:** Performance comparison of frontal face thermal acupoint detection.

Model	MPE (px) ↓	PCK@0.2 ↑	NME (%) ↓	mAP@50–95 ↑	Latency (ms) ↓	Params (M) ↓
Baseline YOLOv8s-Pose	2.024	38.50	2.726	71.56	14.66	12.73
Ours	2.013	39.50	2.709	72.37	11.55	4.64
Relative Improvement	↓ 0.54%	↑ 2.60%	↓ 0.62%	↑ 1.13%	↓ 21.2%	↓ 63.6%

**Table 8:** Performance comparison of profile face thermal acupoint detection.

Model	MPE (px) ↓	PCK@0.2 ↑	NME (%) ↓	mAP@50–95 ↑	Latency (ms) ↓	Params (M) ↓
Baseline YOLOv8n-Pose	3.181	15.24	5.312	86.01	8.57	3.27
Ours	3.145	15.58	4.986	86.41	5.96	1.10
Relative Improvement	↓ 1.13%	↑ 2.23%	↓ 6.13%	↑ 0.46%	↓ 30.4%	↓ 66.3%

**Table 9:** Ablation experiment results of core modules on the frontal face dataset.

Baseline	Remove P5	C2f_SE	Gated FCCR	GeoDeriv	MPE ↓	PCK@0.2 ↑	NME ↓	mAP@50–95 ↑	Latency ↓	Params ↓
✓					2.024	38.50	2.726	71.56	14.66	12.73
✓	✓				2.026	38.45	2.730	71.50	11.20	4.61
✓	✓	✓			2.013	39.50	2.709	72.37	11.55	4.64
✓	✓	✓	✓		1.996	40.15	2.685	73.10	12.10	4.72
✓	✓	✓	✓	✓	1.986	40.58	2.680	73.19	12.10	4.72

**Table 10:** Ablation experiment results of core modules on the profile face dataset.

Baseline	Remove P5	C2f_SE	Gated FCCR	GeoDeriv	MPE ↓	PCK@0.2 ↑	NME ↓	mAP@50–95 ↑	Latency ↓	Params ↓
✓					3.181	15.24	5.312	86.01	8.57	3.27
✓	✓				3.165	15.3	5.25	86.2	5.8	1.08
✓	✓	✓			3.145	15.58	4.986	86.41	5.96	1.1
✓	✓	✓	✓		3.109	16.1	4.905	86.85	6.5	1.13
✓	✓	✓	✓	✓	3.109	16.51	4.895	86.92	6.5	1.13

## Data Availability

The original data presented in the study are openly available in The Tufts Face Database at https://tdface.ece.tufts.edu/ (accessed on 26 December 2024) and Charlotte-ThermalFace: The official github repository at https://github.com/TeCSAR-UNCC/UNCC-ThermalFace (accessed on 26 December 2024).

## Abbreviations

The following abbreviations are used in this manuscript:

2D	Two-dimensional
3D	Three-dimensional
AFDA	Adaptive Frequency Distribution Attention
BN	Batch Normalization
BP	Backpropagation
CLAHE	Contrast Limited Adaptive Histogram Equalization
CTF	Charlotte-ThermalFace
DWConv	Depthwise Separable Convolutions
ECA	Efficient Channel Attention
FCCR	Feature-Conditioned Cascade Refinement
FDPNeck	Frequency-Decoupled Feature Pyramid Neck
FPS	Frames Per Second
GAP	Global Average Pooling
GB/T	Guobiao/Recommended National Standards of the People’s Republic of China
GeoDeriv	Geometric Derivation
GT	Ground Truth
H-Cun	Horizontal Body-Cun
ICAN	Input-Conditional Adaptive Normalization
IN	Instance Normalization
KD	Knowledge Distillation
mAP/MAP	mean Average Precision
ME	Mean Error
MLP	Multilayer Perceptron
MPE	Mean Pixel Error
NCDs	Non-communicable diseases
NIR	Near-infrared
NME	Normalized Mean Error
OKS	Object Keypoint Similarity
PAN	Path Aggregation Network
PCK	Percentage of Correct Keypoints
pp	Percentage points
px	Pixels
RGB	Visible light
ROI	Region of Interest
SE	Squeeze-and-Excitation
SimAM	Simple Attention Module
SPPF	Spatial Pyramid Pooling-Fast
SSD	Single Shot MultiBox Detector
T2FAL	Two-Stage Thermal Facial Acupoint Localization Framework
TAF-IoU	Thermal-Adaptive Focusing IoU
TCM	Traditional Chinese Medicine
TFD	Tufts Face Database
TSG	Thermal Saliency Gate/Thermal Saliency Gating
V-Cun	Vertical Body-Cun
WHO	World Health Organization
YOLO	You Only Look Once
